# Reactivation of p53 by a Cytoskeletal Sensor to Control the Balance Between DNA Damage and Tumor Dissemination

**DOI:** 10.1093/jnci/djv289

**Published:** 2015-10-13

**Authors:** Cecilia Herraiz, Fernando Calvo, Pahini Pandya, Gaia Cantelli, Irene Rodriguez-Hernandez, Jose L. Orgaz, NaRa Kang, Tinghine Chu, Erik Sahai, Victoria Sanz-Moreno

**Affiliations:** **Affiliations of authors:**Tumor Plasticity Laboratory, Randall Division of Cell and Molecular Biophysics, King’s College London, London, UK (CH, PP, GC, IRH, JLO, NK, TC, VSM); Tumor Cell Biology Laboratory, Cancer Research UK London Research Institute, London, UK (FC, ES).; Current affiliations: Tumor Microenvironment Team, Institute of Cancer Research, Chester Beatty Laboratories, London, UK (FC); Department of Biochemistry and Molecular Biology, School of Medicine, University of Murcia and IMIB-Arrixaca, Murcia, Spain (CH).

## Abstract

**Background:**

Abnormal cell migration and invasion underlie metastasis, and actomyosin contractility is a key regulator of tumor invasion. The links between cancer migratory behavior and DNA damage are poorly understood.

**Methods:**

Using 3D collagen systems to recapitulate melanoma extracellular matrix, we analyzed the relationship between the actomyosin cytoskeleton of migrating cells and DNA damage. We used multiple melanoma cell lines and microarray analysis to study changes in gene expression and in vivo intravital imaging (n = 7 mice per condition) to understand how DNA damage impacts invasive behavior. We used Protein Tissue Microarrays (n = 164 melanomas) and patient databases (n = 354 melanoma samples) to investigate the associations between markers of DNA damage and actomyosin cytoskeletal features. Data were analyzed with Student’s and multiple *t* tests, Mann-Whitney’s test, one-way analysis of variance, and Pearson correlation. All statistical tests were two-sided.

**Results:**

Melanoma cells with low levels of Rho-ROCK–driven actomyosin are subjected to oxidative stress-dependent DNA damage and ATM-mediated p53 protein stabilization. This results in a specific transcriptional signature enriched in DNA damage/oxidative stress responsive genes, including Tumor Protein p53 Inducible Protein 3 (TP53I3 or PIG3). PIG3, which functions in DNA damage repair, uses an unexpected catalytic mechanism to suppress Rho-ROCK activity and impair tumor invasion in vivo. This regulation was suppressed by antioxidants. Furthermore, PIG3 levels decreased while ROCK1/2 levels increased in human metastatic melanomas (ROCK1 vs PIG3; *r* = -0.2261, *P* < .0001; ROCK2 vs PIG3: *r* = -0.1381, *P* = .0093).

**Conclusions:**

The results suggest using Rho-kinase inhibitors to reactivate the p53-PIG3 axis as a novel therapeutic strategy; we suggest that the use of antioxidants in melanoma should be very carefully evaluated.

Malignant melanoma is the most serious type of skin cancer because of its high metastatic ability (1–3). Cell migration is a key process during metastatic dissemination of cancer cells. Individual cells can migrate using a variety of strategies, the mesenchymal-”elongated” and the amoeboid-”rounded” modes being the extremes of the spectrum (4–6). Mesenchymal-elongated migration is characterized by actin-dependent protrusions, high adhesion, and lower actomyosin contractility (7,8), while amoeboid migration is driven by high actomyosin contractility (7,8), blebs (9), low adhesion (7,10), and high cytokine signaling (11,12). The contractile cortex is important for amoeboid-rounded to intermediate forms of movement (5,13,14), while some degree of contractility is required to retract protrusions in elongated-mesenchymal migration (15). Therefore, the actomyosin cytoskeleton is key in controlling tumor dissemination.

Rho GTPase signals to ROCK1/2 to promote actomyosin by decreasing myosin phosphatase activity, thus increasing phosphorylation of myosin light chain 2 (MLC2) (16). In migrating cells, Rac and Rho GTPase signaling suppress each other (8,11,14,17,18). The invasive fronts of melanomas are enriched in rounded cells (11,12) with fast amoeboid migration predominating in those invasive fronts (8,11,14,17).

It is unclear how motile cancer cells regulate DNA damage and how this impacts tumor dissemination. Increased generation of reactive oxygen species (ROS) often overcomes the antioxidant systems in cancer cells, resulting in oxidative stress. ROS act as second messenger molecules when present in low amounts, but at higher concentrations ROS can lead to senescence or apoptosis (19). Melanocytes protect the skin from UV irradiation by producing melanin, which renders cells of melanocytic origin particularly sensitive to ROS (20). It is important to better understand how melanomas respond to oxidative stress.

Free radicals cause DNA damage, and the ataxia-telangiectasia mutated (ATM) protein is activated following DNA damage to sense double-strand breaks (21). ROS are also detected by p53 (22), which has an intricate relationship with oxidative stress (23–25). Mitochondria are a major source of intracellular ROS (26): however, less is known about other sources of ROS in cancer. Nonmitochondrial ROS are produced by NADPH oxidase, regulated by Rac1/3 (27–29) through binding to p67phox (30–32) and by 5-lipoxygenase regulated by Rac1 (33).

ROS signaling is very complex, as indicated by the failure of antioxidant therapies. Clinical trials using antioxidants have resulted in higher cancer incidence in the treated groups (34–37), while some chemotherapies increase ROS and offer therapeutic opportunities (38). We explored the links between actomyosin dynamics driving tumor invasion and oxidative stress–induced DNA damage. We studied changes in gene expression and used in vivo intravital imaging to understand how the DNA damage response impacts invasive behavior. We also investigated the associations between markers of DNA damage and actomyosin cytoskeletal features.

## Methods

### Cell Culture

Human melanoma A375P and A375M2 cells were from Prof. Richard Hynes (HHMI, MIT, USA), and SBCL2, WM1361, Skmel23, WM266.4, 501MEL, and Skmel28 were from Prof. Richard Marais (CRUK Manchester Institute). Cells were maintained in DMEM (Gibco) supplemented with 10% fetal calf serum (FCS), 100 μg/mL streptomycin and 60 μg/mL penicillin. RPMI containing 10% FCS was used for WM1361 and SBCL2. Cells were kept in culture for a maximum of three to four passages, and cell phenotypes were verified in every experiment.

### Animal Welfare

All mice were maintained under specific pathogen-free conditions and handled in accordance with the Institutional Committees on Animal Welfare of the UK Home Office (The Home Office Animals Scientific Procedures Act, 1986). All animal experiments were carried out under licence from the Home Office, UK.

### Tumor Xenografts and Imaging

Nude mice were injected subcutaneously with A375M2 cells stably expressing GFP (control = 7 or wild-type-PIG3 = 7). Tumor growth was monitored and measured at the indicated time intervals until the first mouse was used for imaging (end point). Tumor volume was calculated using the formula: π/6 x (w x l x h). When tumors reached visible size (5–9mm in diameter), mice were anesthetized and imaged as described (39). For intravital imaging, seven different regions were imaged simultaneously for two hours for each tumor (approximately 50 µm deep on average). Moving cells were defined as those that moved 10 μm or more for at least 20 minutes. Immunofluorescence experiments in xenografts are described in the Supplementary Materials (available online).

### Confocal Fluorescent and Time-Lapse Microscopy

Cells seeded on top of a thick layer of collagen I were fixed with p-formaldehyde, permeabilized with 0.3% Triton-X 100 (v/v), and blocked with 4% BSA in PBS for 30 minutes. Cells were then incubated with primary antibody (pMLC Ser19, Cell Signaling) and stained with secondary Alexa Fluor-647 anti-rabbit (Life Technologies) and Alexa Fluor 546-phalloidin for F-actin detection (Life Technologies). Multisite bright-field microscopy of cells in 24-well plates containing thick layers of collagen was performed (8). Further details are given in the Supplementary Materials (available online).

### Immunohistochemistry

The high-density melanoma tissue microarrays (Tissue Microarray ME2082b and Tissue Microarray ME208) were from Biomax (Rockville, MD). PIG3 antigen was retrieved by pressure cooker using sodium citrate buffer (10mM Sodium Citrate, pH 6.0) for 15 minutes and detected with mouse monoclonal PIG3 antibody (PIG3; NBP2-01301 Novus Biologicals Inc., 1:100) and the Liquid Permanent Red Chromogen (Dako). Hematoxylin was used to counterstain. Images were blind-scored for PIG3 intensity as negative, low, moderate, and high. Quantification of cell morphology in Figure 8G was performed on hematoxylin and eosin (H&E) stainings. Each core sample was imaged for three separate fields, and 10 representative cells were scored for roundness index. Details on antibodies, inhibitors, plasmids, transfection, RNAi experiments, expression constructs, tracking and migration assays, manual classification of cell morphology, quantitative real-time one step polymerase chain reaction (PCR), immunoblotting, pull-down assays, quantitative assay of SA-β-gal, intracellular measurement of ROS, Annexin V apoptotic assay, and RNAI sequences are given in the Supplementary Materials (available online).

### Analysis of PIG3, ROCK1, and ROCK2 Expression From Human Databases

Gene expression data of a total of 354 human melanoma samples from The Cancer Genome Atlas (TCGA) database (http://cancergenome.nih.gov/) were used to analyze PIG3, ROCK1, and ROCK2 expression in melanoma progression. We only took account patients who had not received neo-adjuvant treatment prior to the resection of the tumor that yielded the sample submitted for TCGA. We only considered samples with greater than 70% tumor cell content. Normalized mRNA expression data and z-scores for mRNA expression data were downloaded from cBioPortal (40,41) and analyzed as described below.

### Statistical Analysis

Unpaired two-tailed Student’s *t* test, Mann-Whitney’s test, one-way analysis of variance (ANOVA) with Tukey’s post hoc test (for multiple comparisons), multiple *t* test using the Holm-Sidak method, and Pearson correlation were performed using GraphPad Prism (GraphPad Software, San Diego, CA, www.graphpad.com). Two-sided log-rank test was performed using SPSS Statistics (IBM). Data were presented as mean ± standard deviation. Survival curves were estimated based on the Kaplan-Meier method and compared using log-rank test. *P* values were calculated using two-sided tests. *P* values of less than .05 were considered statistically significant.

## Results

### Actomyosin Contractility and Reactive Oxygen Species

**Figure 1.**
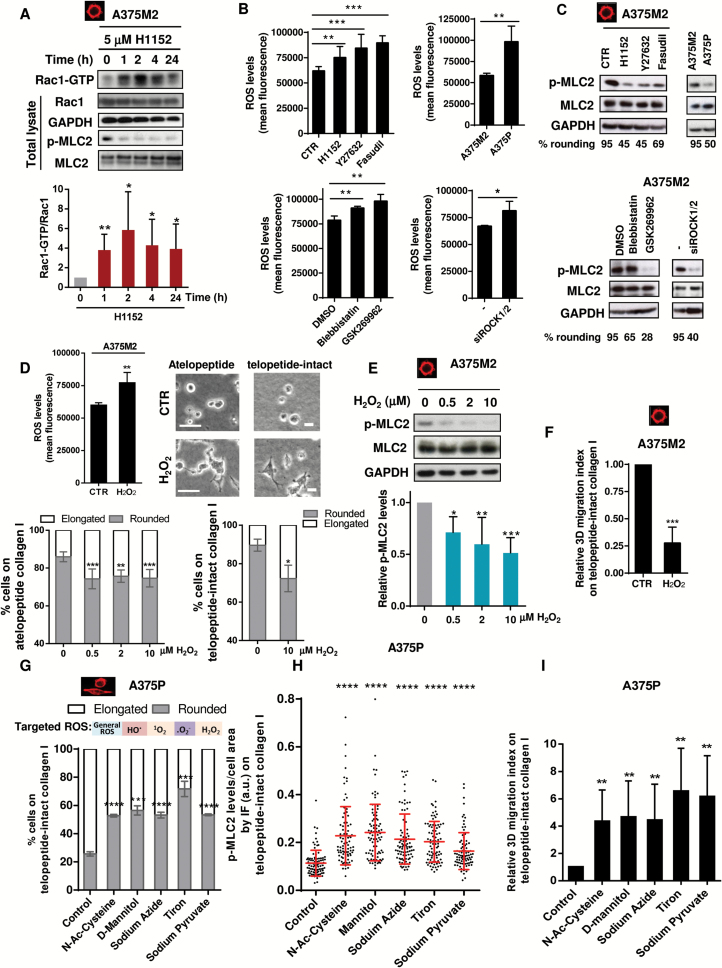
Actomyosin contractility and oxidative stress. **A**) A375M2 cells were treated with 5 μM H1152 for different times from one hour to 24 hours, and Rac1 activation was assessed by pulldown. Representative immunoblot (**top**) and quantification (**bottom**) of Rac1-GTP in pulldown samples and of total Rac1 in total lysate (n = 6, **error bars** are ±SD, two-sided Student’s *t* test was used to generate *P* values, *P < .05, **P < .01). **B**) ROS measurement of A375M2, ROCK-inhibited A375M2 (H1152, 5 μM, Y27632, 10 μM, Fasudil, 10 μM, and GSK269962, 1 μM), A375P cells, contractility-inhibited A375M2 (blebbistatin, 20 μM), and ROCK1/2-depleted A375M2 cells after incubation with DCFH-DA (20 μM). Values are represented as relative mean fluorescence (n = 5, **error bars** are ±SD, two-sided Student’s *t* test was used to generate *P* values, *P < .05, **P < .01, ***P < .001). **C**) Representative immunoblots of myosin light chain II phosphorylation (p-MLC2) levels of cells (n = 5). **D**) ROS measurement of A375M2 cells treated with 10 μM H_2_O_2_ for 24 hours is shown in the **top left panel**. CTR = control (n = 5, **error bars** are ±SD, two-sided Student’s *t* test was used to generate *P* values, **P < .01). **Top right panel**: representative bright-field images of A375M2 cells on top of atelopeptide bovine and telopeptide-intact rat tail collagen I incubated with 10 μM H_2_O_2_ for 24 hours. **Scale bar** = 20 μm. **Bottom left panel**: cell morphology A375M2 cells on top of atelopeptide bovine collagen I treated with increasing concentrations of H_2_O_2_ for 24 hours (n = 6, **error bars** are ±SD, two-sided one-way analysis of variance (ANOVA) with Tukey′s post hoc test was used to generate *P* values, **P < .01, ***P < .001). **Bottom right panel**: cell morphology A375M2 cells on telopeptide-intact collagen I incubated with 10 μM H_2_O_2_ for 24 hours (n = 3, **error bars** are ±SD, two-sided Student’s *t* test was used to generate *P* values, *P < .05). **E**) Representative immunoblot (**top**) and quantification (**bottom**) of MLC2 phosphorylation after treatment with increasing concentrations of H_2_O_2_ for 24 hours (n = 7, **error bars** are ±SD, two-sided one-way ANOVA with Tukey’s post hoc test was used to generate *P* values, *P < .5, **P < .01, ***P < .001). **F)** 3D migration into telopeptide-intact rat tail collagen I of A375M2 cells after stimulation with 10 μM H_2_O_2_ (n = 5, **error bars** are ±SD, two-sided Student’s *t* test was used to generate *P* values, ***P < .001. **G**) Cell morphology of A375P cells treated with the ROS scavengers N-acetyl cysteine, mannitol, sodium azide, tiron, and sodium pyruvate for 24 hours on top of telopeptide-intact rat tail collagen I (n = 3, **error bars** are ±SD, two-sided Student’s *t* test was used to generate *P* values, ***P < .001, ****P < .0001). **H**) Quantification of p-MLC2 fluorescence signal relative to the cell area of confocal images in A375P cells after incubation with the ROS inhibitors N-acetyl cysteine, mannitol, sodium azide, tiron, and sodium pyruvate on telopeptide-intact rat tail collagen I. **Dots** represent single cells from three independent experiments, and values are normalized to the cell area (n = 3 experiments; N = 90 cells, **error bars** are ±SD, two-sided one-way ANOVA with Tukey’s post hoc test was used to generate *P* values, ****P < .0001). **I**) 3D migration into telopeptide-intact rat tail collagen I of A375P cells treated with ROS scavengers N-acetyl cysteine, mannitol, sodium azide, tiron, and sodium pyruvate (n = 6, **error bars** are ±SD, two-sided Student’s *t* test was used to generate *P* values, **P < .01). See also Supplementary Figure 1 (available online).

The actomyosin cytoskeleton favors fast migration in vivo. Therefore, we wanted to understand how cells with lowered actomyosin contractility adapt to cytoskeletal changes. Rac is crucial for the NADPH oxidase complex that generates H_2_O_2_ (42). Through aquaporins (43,44) or passive diffusion (45,46), H_2_O_2_ gets internalized, generating oxidative stress (47). We previously described how decreasing actomyosin contractility increased Rac1 activity after one to two hours of treatment (8). Using longer treatments, we found that Rac1 activation is sustained for 24 hours (*P =* .037) after ROCK inhibitor treatment (Figure 1A). We compared rounded-contractile A375M2 melanoma cells with more elongated ROCK-inhibited A375M2 cells (treated with H1152, Y27632, or Fasudil for 24 hours) (*P =* .0047, *P <* .001, *P <* .001) or with intrinsically less contractile A375P cells, with higher Rac1 activity (7,8) (Figure 1, B and C). We observed an increase in ROS in all conditions leading to lower contractility. These results were confirmed using blebbistatin, ROCK inhibitor GSK269962, or RNAi against ROCK1/2 (*P =* .023) (Figure 1, B and C, lower panels). Furthermore, after ROCK inhibition the increase in ROS levels was dependent on Rac1 (Supplementary Figure 1A, available online).

The inter-relationship between ROS is shown in Supplementary Figure 1B (available online). The addition of H_2_O_2_ to A375M2 cells resulted in increased intracellular ROS (*P =* .0075) (Figure 1D, left) and loss of cell rounding using different types of collagen I (*P =* .033) (Figure 1D, top right panel and bottom panels). These morphological changes were associated with decreased p-MLC2 (Figure 1E), increased Rac1-GTP (Supplementary Figure 1C, available online), and decreased 3D migration (*P <* .001) (Figure 1F). Conversely, the addition of ROS scavengers to A375P cells resulted in increased percentage of rounded cells in different types of collagen (Figure 1G; Supplementary Figure 1, D and E, available online). We could also measure an increase in p-MLC2 (12) (Figure 1H; Supplementary Figure 1, F and G, available online), increased blebbing (Supplementary Figure 1G, available online), and increased 3D migration (Figure 1I). These data show an inverse association between oxidative stress and “amoeboid” invasion.

### Actomyosin Contractility and DNA Damage

We previously performed microarray analysis for gene expression changes positively associated with actomyosin contractility (11), where contractility of A375M2 cells was lowered with two ROCK inhibitors (H1152 or Y27632) or blebbistatin. We re-analyzed this gene dataset using Gene Set Enrichment Analysis (GSEA) and focused on upregulated genes that are enriched in low contractile cells. We found upregulation of genes involved in ROS metabolism and DNA damage responses (Supplementary Figure 2A and Supplementary Table 1, available online). We found similar transcriptional responses comparing A375M2 cells with A375P, intrinsically less contractile (8,11,48) (Supplementary Table 2, available online).

**Figure 2.**
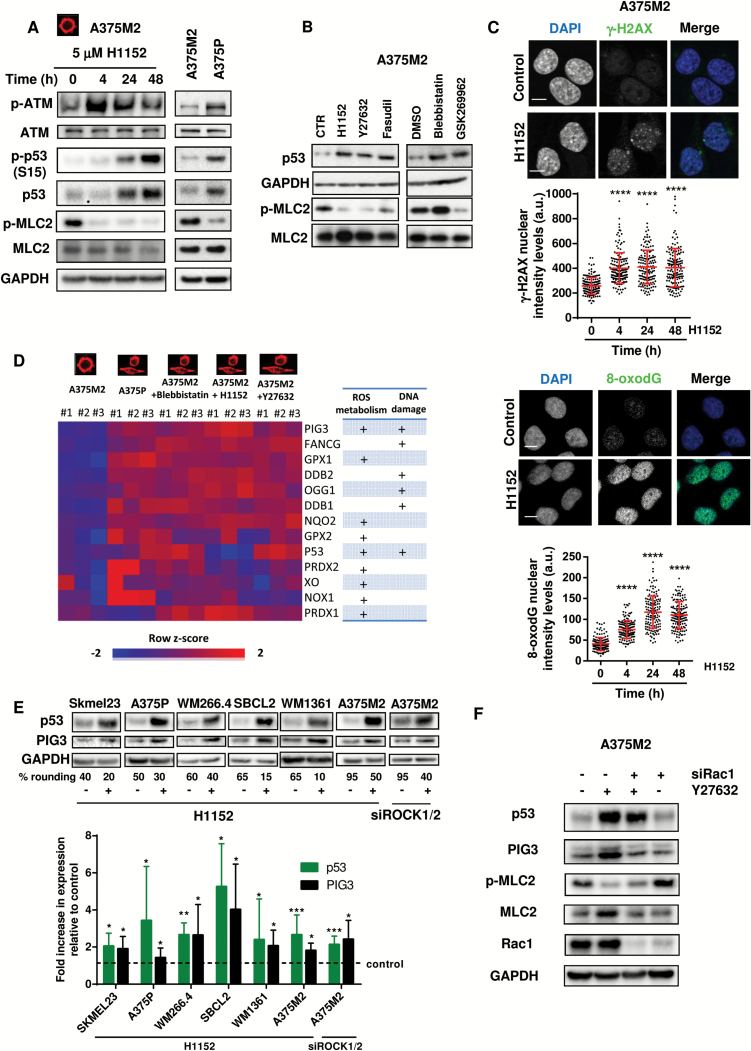
Actomyosin contractility and DNA damage. **A**) Immunoblots for p-ATM, p-p53 (S15), p53, and p-MLC2 of A375M2 and A375P cells treated with ROCK inhibitor H1152 (5 μM) for the indicated time points (n = 5, representative blots are shown). **B**) Immunoblots for p53 and p-MLC2 levels of A375M2 cells treated with ROCK inhibitors H1152 (5 μM), Y27632 (10 μM), Fasudil (10 μM), and actomyosin inhibitor blebbistatin (20 μM) and ROCK inhibitor GSK269962 (1 μM) (n = 5, representative blots are shown). **C) Top panel**: representative confocal images (**top**) of γ-H2AX (**green**) immunostaining in A375M2 cells treated with ROCK inhibitor H1152. DAPI was used to stain DNA (**blue**). **Scale bar** = 10 μm. Quantification of the nuclear levels in arbitrary units (a.u.) of γ-H2AX in A375M2 upon ROCK inhibition (H1152) from three independent experiments is shown below (n = 3 experiments; N = 150 nuclei, **error bars** are ±SD, two-sided one-way analysis of variance (ANOVA) with Tukey’s post hoc test was used to generate *P* values, ****P < .0001). **Bottom panel**: representative confocal images (**top**) of 8-oxodG (**green**) immunostaining in A375M2 cells treated with ROCK inhibitor H1152. DAPI was used to stain DNA (**blue**). **Scale bar** = 10 μm. Quantification of the nuclear levels in arbitrary units (a.u.) of 8-oxodG in A375M2 upon ROCK inhibition (H1152) from three independent experiments is shown below (n = 3 experiments; N = 150 nuclei, **error bars** are ±SD, two-sided one-way ANOVA with Tukey’s post hoc test was used to generate *P* values, ****P < .0001). **D**) Heat map of ROS-regulating gene expression following inhibition of actomyosin contractility in A375M2 cells—conditions shown are A375M2 cells, A375P cells, A375M2 cells treated with blebbistatin, H1152, or Y27632. **Blue** indicates under expression, **red** overexpression, and **intensity of color** indicates relative change. **Rows** were colored using a z-score derived from a gene’s expression across all samples (row z-score). A **table** indicating the involvement of these genes in ROS metabolism and/or DNA damage is shown on the right. **E**) Representative immunoblots (**top**) and quantification (**bottom**) of p53 and PIG3 levels in human wild-type TP53 melanoma Skmel23 (n = 5), A375P (n = 10), WM266.4 (n = 5), SBCL2 (n = 5), WM1361 (n = 13), and A375M2 cells after treatment with ROCK inhibitor H1152 (5 μM) for 24 hours (n = 10) or after abrogation of ROCK1 and ROCK2 with siRNA for 72 hours (n = 7) in A375M2 cells (**error bars** are ±SD, two-sided Student’s *t* test was used to generate *P* values, *P < .05, **P < .01, ***P < .001). **F**) Representative immunoblots for p53, PIG3, p-MLC2, and Rac1 of A375M2 cells after depletion of Rac1 for 72 hours and ROCK inhibition treatment with Y27632 (10 μM) for 24 hours (n = 4). See also Supplementary Table 1 and 2 and Supplementary Figure 2 (available online).

Following ROS generation, DNA damage causes ATM autophosphorylation, which phosphorylates p53 and protects it from degradation (21,49,50). After ROCK inhibition using H1152, we could detect p-ATM (*P =* .041, 24 hours) and p-p53 increases (*P =* .02, 24 hours) (Figure 2A; Supplementary Figure 2B, available online), resulting in p53 stabilization. The same results were observed comparing high-contractile A375M2 with low-contractile A375P (Figure 2A; Supplementary Figure 2C, available online). Other ROCK inhibitors (Y27632, Fasudil, and GSK269962) and blebbistatin also stabilized p53 protein (Figure 2B; Supplementary Figure 2D, available online). ATM promotes H2AX phosphorylation (γ-H2AX), which can be detected in DNA Repair foci (51). We could measure increased p-H2AX in the nucleus (*P <* .0001 all doses) (Figure 2C top panel; Supplementary Figure 2E, available online) and increased 8-hydroxy-2’- deoxyguanosine (8-oxodG) staining (*P <* .0001 all doses) (Figure 2C bottom panel; Supplementary Figure 2F, available online).

We selected genes that were upregulated as a result of ROS-induced DNA damage and are associated with lower actomyosin levels. We found an upregulation of genes involved in ROS production—TP53I3 (PIG3), TP53, NOX1, XDH, and NQO2 (Figure 2D)—and ROS detoxification—GPX and PRDX—indicating that cells are sensing/responding to oxidative stress. Some genes have DNA binding activity as a result of DNA damage—DDB1/2, OGG1, FANCG, and TP53I3 (PIG3). Importantly, p53 and PIG3 were involved both in DNA damage and ROS and were inversely associated with actomyosin contractility (Figure 2D). PIG3 is a component of the DNA damage response pathway that recruits DNA repair machinery to DNA breaks (52) but also harbours oxido-reductase activity (53). We therefore focused on studying p53-PIG3 and its relationship with actomyosin.

Similar to p53, PIG3 was induced after ROCK inhibition (Supplementary Figure 2G, available online). We used a panel of melanoma cells with varying degrees of rounding (11) and wild-type p53. Lowering actomyosin contractility using ROCK inhibitor or via RNAi resulted in loss of cell rounding and increased p53 and PIG3 levels (Figure 2E). Similar results were obtained with ROCK inhibitor Y27632 (Supplementary Figure 2H, available online). Rac1 depletion rescued the p53-PIG3 increase after lowering actomyosin contractility (Figure 2F). These results show that p53-PIG3 expression is dependent on Rac1. This indicates that low actomyosin contractility promotes gene expression programs involved in ROS metabolism and DNA damage response.

### 3D Migration and PIG3 in Melanoma

**Figure 3.**
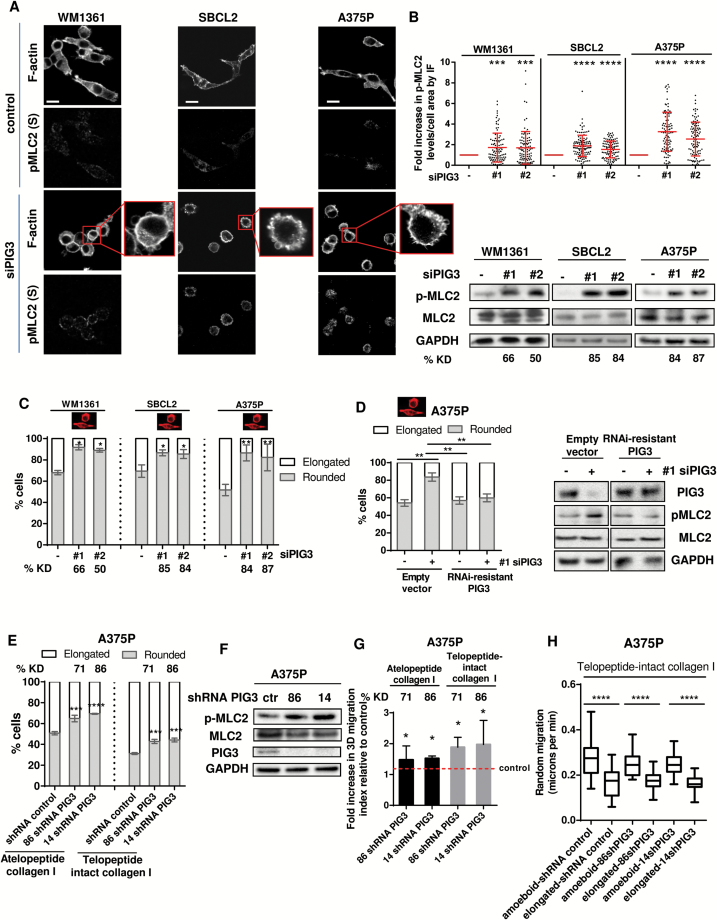
3D migration and PIG3 in melanoma. **A**) Representative confocal images of p-MLC2 (Ser19) and F-actin immunostaining in PIG3-depleted WM1361, SBCL2, and A375P cells on atelopeptide bovine collagen I. **Scale bar** = 20 μm. Inset shows cell blebbing in PIG3-depleted cells (n = 3, representative blots are shown). **B**) Quantification of p-MLC2 immunofluorescence (IF) signal relative to the cell area of confocal images in PIG3-abrogated melanoma cells WM1361, SBCL2, and A375P. Each **dot** represents a single cell from three independent experiments (n = 3 experiments; N = 105 cells, error bars are ±SD, two-sided one-way analysis of variance (ANOVA) with Tukey’s post hoc test was used to generate *P* values, *P < .05, ***P < .001, ****P < .0001). Immunoblots for p-MLC2 levels in PIG3-ablated WM1361, SBCL2, and A375P cells seeded on collagen I are shown on the **right**. Percentage of PIG3 knockdown (KD) is shown below. **C**) Cell morphology of WM1361, SBCL2, and A375P cells on atelopeptide bovine collagen I after PIG3 depletion. Two individual On Target (OT) siRNA oligonucleotides were transfected to deplete PIG3 expression (#1 and #2). PIG3 knockdown (KD) levels are shown below as % (n = 3 in WM1361 and SBCL2 and n = 4 in A375P, **error bars** are ±SD, two-sided one-way ANOVA with Tukey’s post hoc test was used to generate *P* values, *P < .05, **P < .01). **D) Left panel**: cell morphology of A375P cells overexpressing empty vector or RNAi resistant PIG3 after PIG3 knockdown (n = 3, **error bars** are ±SD, two-sided Student’s *t* test was used to generate *P* values, **P < .01). Representative immunoblots for PIG3 and p-MLC2 levels are shown on the **right panel** (n = 3, representative blots are shown). **E**) Cell morphology of A375P cells stably transfected with shRNA for PIG3 and seeded on atelopeptide bovine and telopeptide-intact rat tail collagen I (n = 3, **error bars** are ±SD, two-sided one-way ANOVA with Tukey’s post hoc test was used to generate *P* values, ***P < .001, ****P < .0001). Percentage of PIG3 knockdown (KD) is shown above. **F**) Representative immunoblots of p-MLC2 and PIG3 levels of A375P cells on atelopeptide bovine collagen I matrix after shRNA depletion of PIG3 (n = 3, a representative experiment is shown). Ctr stands for control. **G**) 3D migration into atelopeptide bovine and telopeptide-intact rat tail collagen I of A375P cells after PIG3 depletion with shRNA (n = 5, **error bars** are ±SD, two-sided one-way ANOVA with Tukey’s post hoc test was used to generate *P* values, *P < .05). Percentage of PIG3 knockdown (KD) is shown above. **H**) Random migration of PIG3-depleted A375P cells on telopeptide-intact collagen I (n = 3 experiments; N = 30 cells, **error bars** are ±SD, two-sided Student’s *t* test was used to generate *P* values, ****P < .0001). See also Supplementary Figure 3 and Supplementary Movies 1 and 2 (available online).

We then depleted PIG3 in several melanoma cell lines using different RNAi (Figure 3, A-C; Supplementary Figure 3A, available online). Depletion of PIG3 resulted in increased p-MLC2 using immunofluorescence (Figure 3, A and B, top panel) and immunoblot (Figure 3B, bottom panel) and increased percent rounded cells (Figure 3C; Supplementary Figure 3, A and B, available online). Similar results were observed after p53 depletion, which resulted in PIG3 downregulation (Supplementary Figure 3, C and D, available online). Overexpression of RNAi-resistant PIG3 rescued these effects (*P =* .003) (Figure 3D; Supplementary Figure 3E, available online).

We generated stable cell lines using two shRNAs against PIG3. Reducing PIG3 in A375P cells resulted in increased percent rounded cells (Figure 3E; Supplementary Movies 1 and 2, available online), increased p-MLC2 levels (Figure 3F; Supplementary Figure 3F, available online), and increased 3D-migration (Figure 3G). Moreover, cell movement analysis showed that A375P cells using amoeboid migration after PIG3 depletion were statistically significantly faster than A375P cells using elongated migration (*P* < .0001) (Figure 3H). These results show that loss of PIG3 as a result of loss of p53 function favors increased fast rounded-amoeboid behavior.

### PIG3 Catalytic Activity and the Actomyosin Cytoskeleton

**Figure 4.**
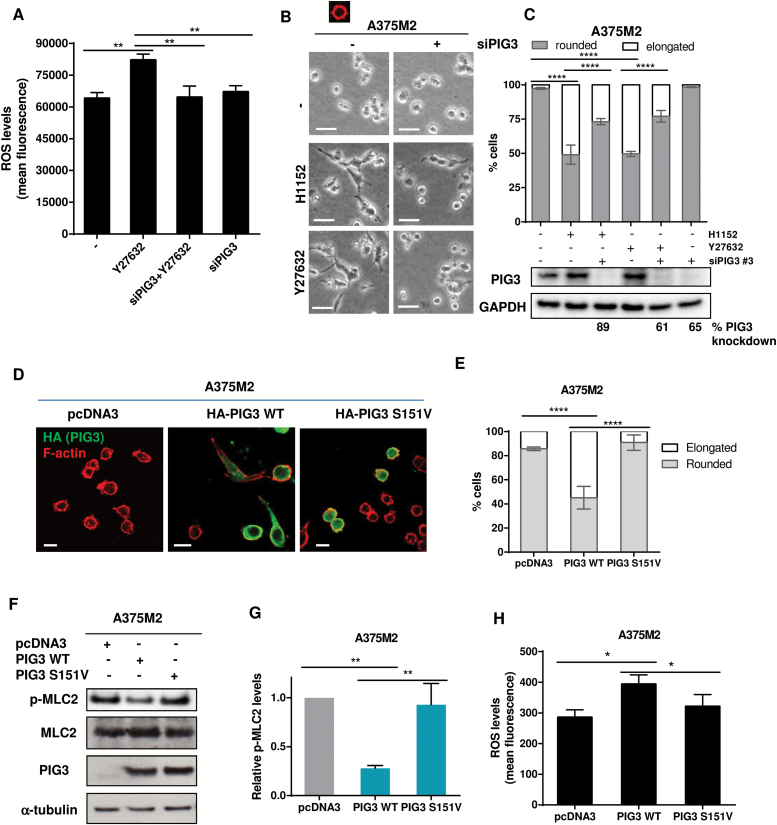
PIG3 catalytic activity and the actomyosin cytoskeleton. **A**) ROS measurements of A375M2 treated with ROCK inhibitor Y27632 (10 μM) for 24 hours after depletion of PIG3 by siRNA. Values are represented as relative mean fluorescence (n = 3, **error bars** are ±SD, two-sided one-way analysis of variance (ANOVA) with Tukey’s post hoc test was used to generate *P* values, **P < .01). **B**) Representative bright-field images of PIG3-depleted–A375M2 cells on bovine collagen I and incubated with ROCK inhibitors H1152 (5 μM) or Y27632 (10 μM) for 24 hours. **Scale bar** = 20 μM. **C**) Cell morphology of A375M2 cells on top of bovine collagen I after PIG3 depletion prior to treatment with H1152 (5 μM) or Y27632 (10 μM) for 24 hours (n = 3, **error bars** are ±SD, two-sided one-way ANOVA with Tukey’s post hoc test was used to generate *P* values, ***P < .001, ****P < .0001). Percentage of PIG3 knockdown is shown below. **D**) Representative confocal images of PIG3 (**green**) immunostaining in A375M2 cells transiently transfected with empty vector pcDNA3 or with the constructs HA-PIG3 WT or HA-PIG3 S151V on bovine collagen I. F-actin was also stained (**red**). **Scale bar** = 20 μm. **E**) Cell morphology of A375M2 cells overexpressing HA-PIG3 WT or HA-PIG3 S151V on bovine collagen I matrix (n = 4, **error bars** are ±SD, two-sided one-way ANOVA with Tukey’s post hoc test was used to generate *P* values, ****P < .0001). **F**) Representative immunoblots for p-MLC2 and PIG3 protein levels in PIG3-overexpressing A375M2 cells (n = 3, representative blots are shown). **G**) Quantification of MLC2 phosphorylation levels normalized to cells transfected with the empty vector (n = 3, **error bars** are ±SD, two-sided one-way ANOVA with Tukey’s post hoc test was used to generate *P* values, **P < .01). **H**) ROS measurement of A375M2 cells transfected with empty vector pcDNA3, PIG3 WT, or S151V PIG3. ROS levels were represented as relative mean fluorescence (n = 4, **error bars** are ±SD, two-sided one-way ANOVA with Tukey’s post hoc test was used to generate *P* values, *P < .05, **P < .001). See also Supplementary Figure 4 (available online).

PIG3 participates in DNA damage responses in the nucleus and produces ROS in the cytoplasm. We explored if the latter function could impact actomyosin contractility. After ROCK inhibition, increased ROS levels were abolished if PIG3 had been depleted from A375M2 cells (Figure 4A). Morphological changes induced by ROCK inhibition (H1152, Y27632) were partially rescued if PIG3 was ablated (*P <* .0001) (Figure 4, B and C).

PIG3 oxido-reductase activity needs NADP (53). PIG3 S151V is unable to bind NADP and produce ROS (53). PIG3 WT overexpression reduced cell rounding (*P <* .0001) (Figure 4, D and E) and p-MLC2 (*P =* .001) in A375M2 cells (Figure 4, F and G) by increasing ROS (*P =* .003) (Figure 4H). In contrast, mutant PIG3 S151V could not increase ROS and could not suppress amoeboid features (Figure 4, D-H).

PIG3 has been involved in cooperative apoptotic responses (54), but overexpressing PIG3 WT or S151V did not induce apoptosis in A375M2 cells (Supplementary Figure 4, available online) compared with adriamycin. These results show that the catalytic activity of PIG3 opposes actomyosin without inducing apoptosis.

### Regulation of Cytoskeletal Dynamics via PIG3 and ARHGAP5/P190RhoB

**Figure 5.**
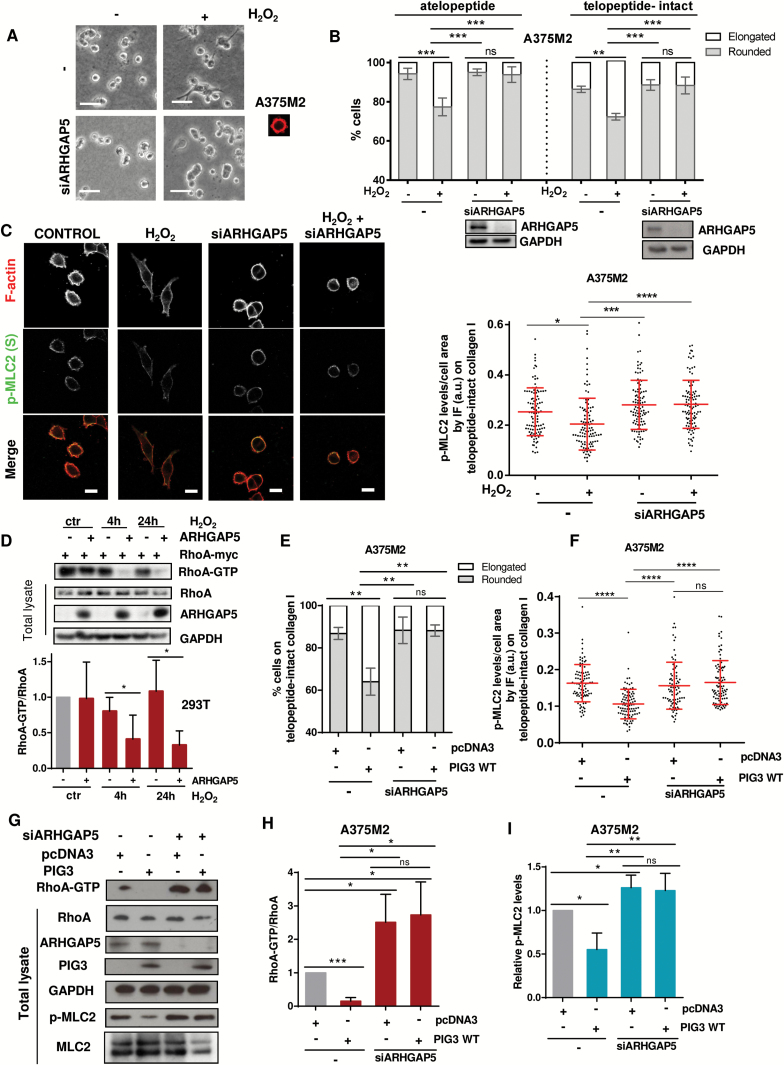
Regulation of cytoskeletal dynamics via PIG3 and ARHGAP5/P190RhoB. **A**) Representative bright-field images seeded on atelopeptide bovine collagen I and 72 hours post-transfection, treated with H_2_O_2_ (10 μM) for 24 hours. **Scale bar** = 20 μm. **B**) Cell morphology of ARHGAP5-depleted A375M2 cells on top of atelopeptide collagen I compared with telopeptide-intact rat tail collagen I after stimulation with 10 μM H_2_O_2_ (n = 3, **error bars** are ±SD, two-sided one-way analysis of variance (ANOVA) with Tukey’s post hoc test was used to generate *P* values, **P < .01, ***P < .001). Immunoblot for ARHGAP5 is shown below. NS = nonsignificant. **C**) Representative confocal images (**left**) of p-MLC2 (**green**) immunofluorescence (IF) and quantification of p-MLC2 (Ser19) levels (**right**) of ARHGAP5-ablated A375M2 cells stimulated with H_2_O_2_ (10 μM) for 24 hours. F-actin was also stained (**red**). Each **dot** represents a single cell; values are relative to the cell area and represented in arbitrary units (a.u.) from three independent experiments (n = 3 experiments; N = 90 cells, **error bars** are ±SD, two-sided one-way ANOVA with Tukey’s post hoc test was used to generate *P* values, *P < .05, ***P < .001, ****P < .0001). **Scale bar** = 20 μm. **D**) Representative immunoblots (**top**) and quantification (**bottom**) showing Myc-RhoA activation and Flag-ARHGAP5 overexpression in HEK293T cells treated with 10 μM H_2_O_2_ (n = 3, **error bars** are ±SD, two-sided Student’s *t* test was used to generate *P* values, *P < .05). **E**) Cell morphology of PIG3-overexpressing A375M2 cells after stimulation with 10 μM H_2_O_2_ (n = 3, **error bars** are ±SD, two-sided one-way ANOVA with Tukey’s post hoc test was used to generate *P* values, ****P < .0001). **F**) Quantification of p-MLC2 (Ser19) levels from confocal images of p-MLC2 immunofluorescence (IF) stainings of PIG3-overexpressing A375M2 cells stimulated with H_2_O_2_ (10 μM) for 24 hours. Each **dot** represents a single cell, and values are represented in arbitrary units (a.u.) from three independent experiments. NS = nonsignificant (n = 3 experiments; N = 90 cells, **error bars** are ±SD, two-sided one-way ANOVA with Tukey’s post hoc test was used to generate *P* values, ****P < .0001). **G**) Representative immunoblots showing RhoA activation, ARHGAP5, PIG3, and p-MLC2 levels in A375M2 cells overexpressing PIG3 WT after knocking down ARHGAP5 (n = 4, a representative blot is shown). **H**) Quantification of Rho-GTP normalized to total Rho levels in PIG3-overexpressing A375M2 cells after depleting ARHGAP5 (n = 4, **error bars** are ±SD, two-sided Student’s *t* test was used to generate *P* values, *P < .05). **I**) Quantification of MLC2 phosphorylation levels from **(G)** (n = 4, **error bars** are ±SD, two-sided Student’s *t* test was used to generate *P* values, *P < .05, **P < .01). See also Supplementary Figure 5 (available online).

GTPase activating proteins (GAPs) are negative regulators of Rho GTPases (55), and generation of ROS can activate p190Rho-GAP family and downregulate Rho (56). Depletion of p190Rho-GAP (p190A) in A375M2 cells did not affect cell morphology (Supplementary Figure 5A, available online). Depletion of p190B (ARHGAP5) (57) rescued morphological changes (*P =* .002) and p-MLC2 changes (*P <* .0001) after H_2_O_2_ addition (Figure 5, A-C; Supplementary Figure 5B, available online). Depletion of ARHGAP5 in A375P and WM1361 cells yielded similar results (Supplementary Figure 5, C-E, available online). Overexpression of Flag-ARHGAP5 in 293T cells treated with H_2_O_2_ for four hours and 24 hours resulted in reduced Rho activity levels (*P =* .043 at 4 hours, *P =* .042 at 24 hours) (Figure 5D). Therefore, the GAP activity of ARHGAP5 is stimulated after an increase in ROS.

**Figure 6.**
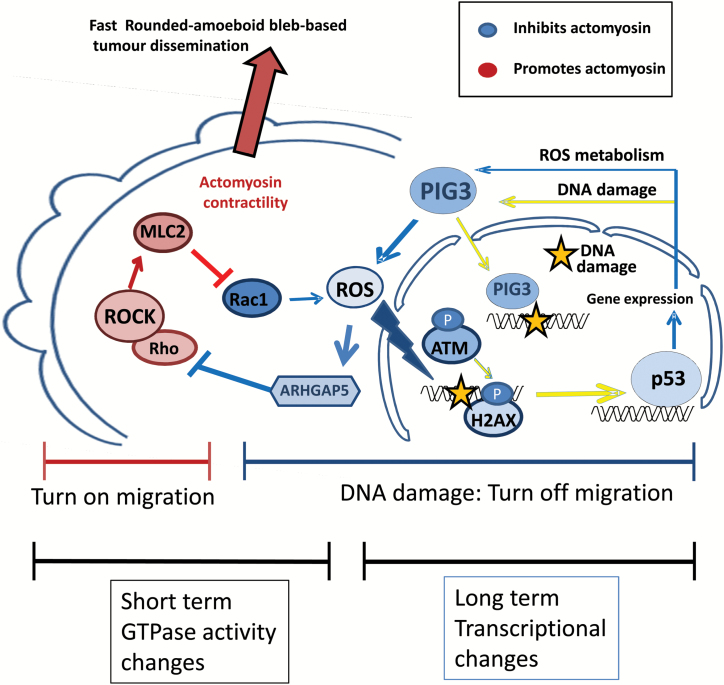
Balance between Rho-ROCK and oxidative stress–induced DNA damage. Oxidative stress–dependent DNA damage results in p53 stabilization in cells with low actomyosin contractility. In turn, p53 drives the expression of several genes involved in oxidative stress metabolism and DNA damage responses, including PIG3. PIG3, apart from its DNA damage response functions, sustains production of ROS to inhibit cytoskeletal Rho-ROCK signaling through ARHGAP5, suppressing rounded-amoeboid fast tumor dissemination.

Overexpression of PIG3 did not alter cell morphology and actomyosin levels if cells had been depleted from ARHGAP5 (Figure 5, E and F; Supplementary Figure 5, F and G, available online). PIG3 overexpression resulted in decreased levels of active RhoA (*P <* .001) (Figure 5, G and H), but a recovery of Rho activity (*P =* .013) and p-MLC2 levels (*P =* .003) was measured after ARHGAP5 depletion (Figure 5, G-I). These results show that PIG3-dependent oxidative stress activates ARHGAP5 to decrease Rho-ROCK-MLC2 signaling. An inverse relationship between actomyosin and oxidative stress–induced DNA damage is established, short term via regulation of GTPase activity and long term via transcriptional changes. Cells repairing oxidative stress driven DNA damage will activate p53-PIG3 and ARHGAP5 to suppress actomyosin contractility–driven migration (Figure 6).

### PIG3 and Cancer Motility In Vivo

**Figure 7.**
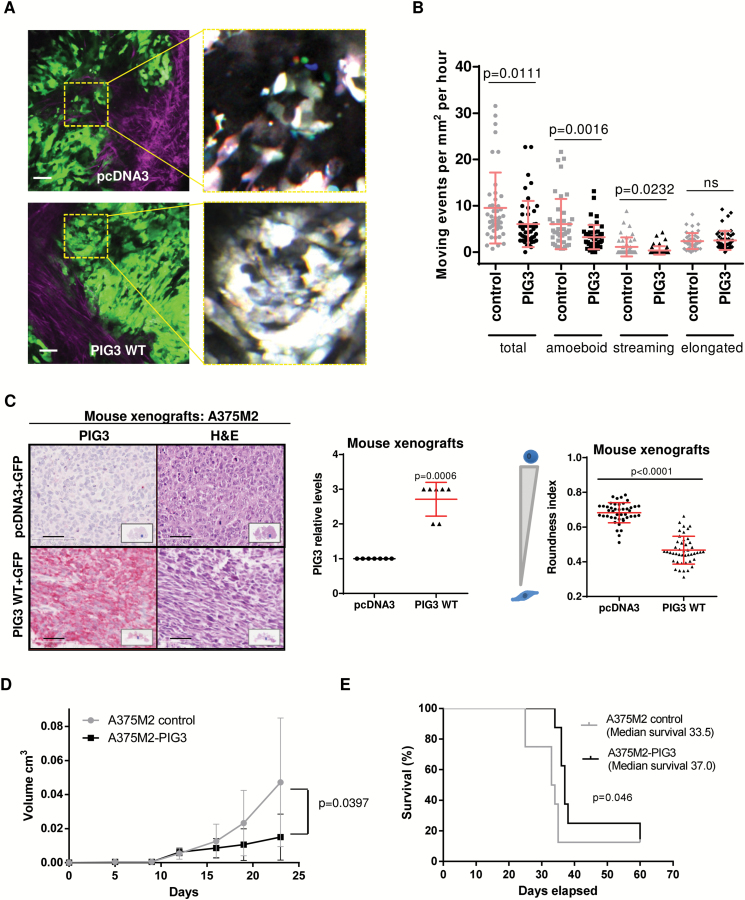
PIG3 and cancer motility in vivo**. A**) Multiphoton intravital microscopy of A375M2 subcutaneous tumors (GFP) and representative images of control (**top**) and PIG3 WT (**bottom**) stably transfected cells. **Right panels** show at higher magnification motion analysis of areas indicated in **yellow**, **red**, **green**, and **blue** images from three time points 630s apart are overlaid, and distinct areas of color indicate motile cells. **Scale bar** = 50 μm. **B**) Quantification of the number of motile cells in control and in PIG3 WT–overexpressing A375M2 cells (7 fields/mouse; 7 mice/condition, **error bars** are ±SD, two-sided Student’s *t* test was used to generate *P* values). **C**) Representative images of mouse xenografts sections of pcDNA3 or PIG3 WT–overexpressing A375M2 stained for PIG3 or for hematoxylin and eosin (H&E). **Scale bar** = 50 μm. **Graph** representing PIG3 expression levels in the xenografts is shown in the middle (7 mouse xenografts/condition, **error bars** are ±SD, two-sided Student’s *t* test was used to generate *P* values). Cell morphology (roundness index) calculated from H&E images of xenografts of mouse tumors of A375M2 control or PIG3 WT–overexpressing cells is shown on the **right panel** (7 mouse xenografts/condition, 6 fields/mouse xenograft, **error bars** are ±SD, two-sided Student’s *t* test was used to generate *P* values). **D**) Volume of subcutaneous tumors at different days after injecting A375M2 or A375M2 cells overexpressing PIG3 WT. The results show mean volumes measured on days 5, 9, 12, 16, 19, and 23 for groups of seven mice/condition, with **error bars** to represent ±SD. A two-sided Student’s *t* test was used to calculate *P* value for day 23. **E**) Survival curves for subcutaneous xenograft mouse models of A375M2 control cells and PIG3 WT–overexpressing A375M2 cells. The survival curve represents the percentage of animals alive at the indicated time point after injection. (Survival curves were estimated by the Kaplan Meier method and compared among subsets using the log-rank test. Differences with a *P* value < .05 were considered statistically significant, and all tests were two-sided).

We next used two-photon intravital imaging to examine the movement of cells in A375M2 xenografts. We generated GFP-stable cell lines where PIG3 was overexpressed (Supplementary Figure 6, A-D, and Supplementary Movies 3 and 4, available online). When tumors reached the same size, imaging showed that tumor cell motility was reduced in PIG3-overexpressing cells compared with control cells (*P =* .011) (Figure 7, A and B; Supplementary Movies 5 and 6, available online). Figure 7A shows merged red, green, and blue images taken from three different time points 630 s apart in the time-lapse movies: spatial separation of colors indicates movement, whereas cells that do not migrate appear white (17). In A375M2 tumors, cell movement at the periphery is predominantly rounded, “amoeboid” movement (8,11,14,17). Melanoma cells can move as single cells or follow the same path (58). Cells following the same paths are using multicellular “streaming” as a mode of motility, whereas cells in isolation are using single-cell motility (58). The reduction in motility of PIG3-overexpressing cells was associated with reduced levels of rounded- “amoeboid” movement and “streaming amoeboid” movement (*P =* .0016, *P =* .023, respectively) (Figure 7B; Supplementary Figure 6E, available online). These data show that PIG3 suppresses “amoeboid” migration both in vitro and in vivo.

See also Supplementary Figure 6 and Supplementary Movies 3–6 (available online).

We confirmed that PIG3-overexpressing cells gave rise to tumors with increased expression of PIG3, as measured by immunohistochemistry (IHC) (*P <* .001) (Figure 7C; Supplementary Figure 6F, available online) and that were associated with decreased cell rounding (*P <* .0001) (Figure 7C, right panel). Such PIG3-overexpressing tumors grew slower than controls (*P =* .0397, day 23) (Figure 7D), and mice survived longer (median survival, days: A375M2 control: 33.5; A375M2-PIG3: 37.0; *P =* .046) (Figure 7E). These results indicate that PIG3 re-expression in melanoma cells suppresses fast amoeboid movement.

### Actomyosin Contractility and PIG3 in Human Melanomas

**Figure 8.**
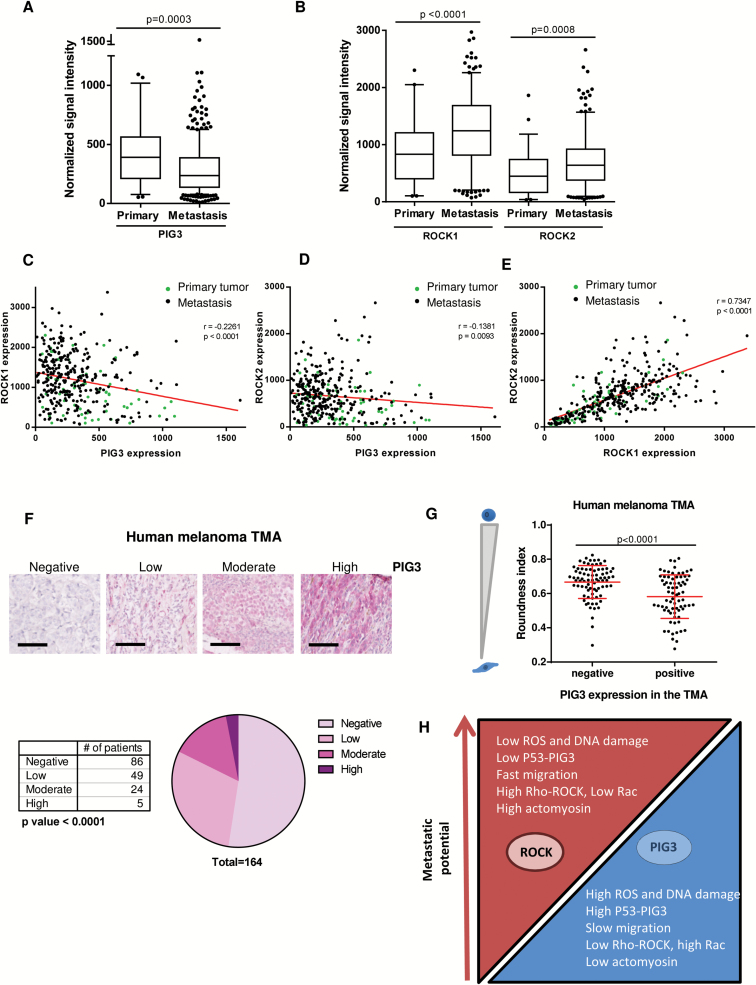
Actomyosin contractility and PIG3 in human melanomas. **A**) PIG3 expression in primary and metastasis melanoma human tissues using normalized mRNA expression data from The Cancer Genome Atlas (TCGA) database. **B**) ROCK1 and ROCK2 expression in primary and metastasis melanoma human tissues using normalized mRNA expression data from TCGA database. **A and B) Box plots** show the median, quartiles, 10% and 90% values and outliers (Mann-Whitney test was used to generate *P* values). **C-E**) Scatter plot of PIG3, ROCK1, and ROCK2 expression correlation analysis (Pearson’s r) using normalized mRNA expression data from TCGA database. **C and D**) Results showed a significant negative correlation between PIG3 and ROCK1 expression (r = -0.2261) and between PIG3 and ROCK2 expression (r = -0.1381). **E**) Analysis revealed a significant positive correlation between ROCK1 and ROCK2 expression (r = 0.7347). **F**) Number of melanoma patients expressing negative, low, moderate, or high levels of PIG3 in the tissue microarrays (TMAs). The number of melanoma patients expressing the different PIG3 levels is shown in the **graph** (Chi-square test was used to generate *P* values). A representative image of the four grades of staining intensity used to score PIG3 expression in the tissue microarray is shown above. **Scale bar** = 100 μm. **G**) Association between morphological features (roundness index) and PIG3 staining intensity in two melanoma TMAs. Stain intensity was scored as negative or positive (low, moderate, and high level) intensity for PIG3 expression in the TMAs. For cell morphology, three random fields per core in the hematoxylin and eosin–stained TMAs were randomly chosen and the roundness index was calculated using ImageJ (30 cells/core) (**error bars** are ±SD, two-sided Student’s *t* test was used to generate *P* values). **H**) Diagram representing the relationship between cytoskeletal Rho-ROCK signaling, PIG3 catalytic activity resulting in high oxidative stress-dependent DNA damage and its association with less tumor invasion, and thus less metastatic potential. See also Supplementary Figure 7 (available online).

We used the publicly available database TCGA (http://cancergenome.nih.gov/) and found that PIG3 levels were decreased in metastatic lesions (*P <* .001), while the actomyosin regulators ROCK1 and ROCK2 were increased (*P <* .0001 ROCK1, *P <* .001 ROCK2) (n = 354) (Figure 8, A and B; Supplementary Figure 7A, available online). There was a negative correlation between PIG3 and ROCK1/2 levels (*r* = -0.2261, *P <* .0001 ROCK1 vs PIG3, *r* = -0.1381 *P =* .0093 ROCK2 vs PIG3) (Figure 8, C and D; Supplementary Figure 7B, available online) and a positive correlation between ROCK1 and ROCK2 (*P <* .0001) (Figure 8E; Supplementary Figure 7B, available online).

We then used IHC in tissue microarrays (TMAs) (n = 164 melanoma patients) to associate PIG3 expression with morphological features. Each sample was scored blind for intensity as negative (0), low (1), moderate (2), or high (3) (Figure 8F). Fifty percent of human melanoma patients had lost PIG3 protein expression, while 30% had low expression and 3% retained high PIG3 expression (*P <* .0001) (Figure 8F). PIG3 levels were negatively associated with cell rounding in human melanomas (*P <* .0001) (Figure 8G). These results show that the suppressive mechanism that PIG3 exerts on invasive behavior is frequently lost in melanomas and it associates with a gain in actomyosin (Figure 8H).

## Discussion

We described an inverse balance between actomyosin contractility and oxidative stress-induced DNA damage (Figure 6 and Figure 8H). These results suggest that antioxidants will favor actomyosin contractility and invasive behavior. Importantly, the subsequent DNA damage response will suppress the actomyosin machinery to impair migration of DNA-damaged cells. Melanoma cells have a fine-tuned internal sensor regulating the balance between DNA damage/DNA repair and actomyosin contractility, which will determine if cancer cells will spread.

In melanoma, p53 mutations are less frequent than in other skin cancers (59–62), but functional attenuation is needed for melanoma development (63). New approaches to reactivate p53 for melanoma therapy are needed (64,65). We find enhanced p53 when actomyosin contractility is lowered. RhoC is a key regulator of melanoma metastasis (48), and high actomyosin levels driven by Rho-ROCK activity could impair some p53 functions in late stages of melanoma. In line with this, we found an inverse correlation between ROCK1/2 and PIG3 levels in melanoma.

Interestingly, we found that PIG3 expression is positively regulated by Rac1-induced ROS production via p53 stabilization (Figure 2F). We describe an alternative mechanism by which Rac1 suppresses actomyosin contractility via transcriptional regulation of PIG3. In the long term, PIG3 upregulation could be supporting and sustaining ROS production. Upon lowering actomyosin contractility, we observed an upregulation of other genes that could be increasing ROS in the long term, such as NOX1 and XDH. In the short term, Rac1 generation of ROS—via p67phox—could directly activate ARHGAP5 and suppress Rho activity. These two mechanisms are superimposed over time and responsible for the short- and long-term suppression of the actomyosin machinery by Rac1-induced oxidative stress (Figure 6).

PIG3 was discovered with 12 other proteins induced by p53 before apoptosis onset; however, none of these genes was sufficient to induce apoptosis on its own (54). PIG3 has also been reported to recruit Rad50, Mre11, 53BP1, and Nbs1 to sites of DNA break lesions (66). We found that cytoplasmic PIG3 has an important additional and unexpected role in impairing migration of cells that are repairing DNA. It would be interesting to establish if other components of the DNA repair machinery that are upregulated in our study could be relevant in controlling tumor dissemination.

PIG3 expression is regulated through its promoter (67) and is found in most vertebrates, plants, protists, and bacteria, but has not been found in rodents (53). Therefore, melanoma mouse models studying p53 function (63,68–70) may have missed the role of PIG3. In this study, we found that high levels of PIG3 impair tumor invasion by suppressing actomyosin contractility. This reinforces the value of ROCK inhibition to reactivate p53-PIG3 as we observed that combining chemotherapeutics with ROCK inhibition resulted in synergic p53-PIG3 reactivation (Supplementary Figure 8A, available online). These findings could be exploited in the clinic because ROCK inhibitors are well tolerated in humans and used for several disorders (71–73).

One limitation of our study was that we did not distinguish between different intracellular sources of ROS, such as mitochondria, peroxisomes, endoplasmic reticulum, cell membranes, etc. (74). Furthermore, while our study was carried out in p53 wild-type melanomas, mutant-p53 cell lines did not regulate p53 levels upon ROCK inhibition (Supplementary Figure 8B, available online). Mutant p53 displays constitutive p-S15 (75), a key residue protecting p53 from degradation; this could explain why we cannot detect further stabilization. It will be important to determine how DNA repair and tumor migration are integrated in p53-mutant melanomas.

Oncogenic signaling in melanoma leads to the accumulation of high ROS leading to senescence (76) that can be prevented by antioxidants (77,78). Melanocyte senescence and apoptosis can be caused by an imbalance between ROS production and detoxification (20). It will be important to assess if actomyosin controls senescent and apoptotic responses. PIG3-overexpressing cells showed increased enzymatic activity of β-galactosidase over time (Supplementary Figure 8C, available online). Because PIG3-overexpressing tumors grew more slowly, further work is needed to understand if senescence is supported by PIG3.

In the current study, we present an important and intriguing example where increased ROS might be beneficial to suppress tumor invasion; therefore, the broad use of antioxidants should be carefully assessed in melanoma.

## Funding

The work was supported by Cancer Research UK (CRUK) C33043/A12065 (VSM, IRH, JLO); Royal Society RG110591 (VSM). CH is supported by a Federation of European Biochemical Societies fellowship, PP by King’s Overseas Scholarship, GC by Medical Research Council C97993H. IRH by Fundacion Alfonso Martin Escudero. ES and FC are supported by CRUK.

## Supplementary Material

Supplementary Data

## Notes

We thank Cecilia Osorio, Nicole Taylor, and Claire Wells for technical advice. We thank the BRC Flow Cytometry Core at Guy’s Hospital (National Institute for Health Research) for equipment and advice. The views expressed are those of the author(s) and not necessarily those of the NHS, the NIHR or the Department of Health. We thank Anne Ridley for Microscopy facilities.

Author contributions: VSM was the principal investigator who designed the study, coordinated the project, contributed to experiments, and wrote the manuscript; CH performed most of the experiments and contributed to the design and writing of the manuscript; GC, PP, JLO, IRH, NK, and TC contributed to some experiments; ES and FC performed intravital imaging experiments.
