# Supplementary material for: Reactivation of p53 by a Cytoskeletal Sensor to Control the Balance Between DNA Damage and Tumor Dissemination
Source: J Natl Cancer Inst. 2015 Oct 13;108(1):djv289. doi: 10.1093/jnci/djv289 (PMC4712681; doi:10.1093/jnci/djv289)
Supplement: Supplementary Data [file supp_108_1_djv289__index.html]

Reactivation of p53 by a Cytoskeletal Sensor to Control the Balance Between DNA Damage and Tumor Dissemination — Supplementary Data 

# Reactivation of p53 by a Cytoskeletal Sensor to Control the Balance Between DNA Damage and Tumor Dissemination

## Supplementary Data

Data files

- Supplementary Data - Supplementary Data
- Supplementary Data - Supplementary Data
- Supplementary Data - Supplementary Data
- Supplementary Data - Supplementary Data
- Supplementary Data - Supplementary Data
- Supplementary Data - Supplementary Data
- Supplementary Data - Supplementary Data
