# Supplementary material for: Reactivation of p53 by a Cytoskeletal Sensor to Control the Balance Between DNA Damage and Tumor Dissemination
Source: J Natl Cancer Inst. 2015 Oct 13;108(1):djv289. doi: 10.1093/jnci/djv289 (PMC4712681; doi:10.1093/jnci/djv289)
Supplement: Supplementary Data [file supp_djv289_Supplementary_Materials.pdf]

SUPPLEMENTARY MATERIALS

Supplementary Methods

Antibodies and inhibitors

Phospho-MLC2 (rabbit polyclonal, 1:100 dilution, #3671 and rabbit polyclonal, 1:1,000 dilution #3674), RhoA (rabbit monoclonal, 1:1,000 dilution, #2117), phospho-p53 (rabbit polyclonal, 1:1,000 dilution, #9284P) and ARHGAP5 (rabbit polyclonal, 1:1,000 dilution, #2562) from Cell Signaling Technology. MLC2 (rabbit polyclonal, 1:1,000 dilution, sc-15370), ATM (mouse monoclonal, 1:1,1000 dilution, sc-23921), phospho-ATM (mouse monoclonal, 1:1,000 dilution, sc-47739) and p53 (mouse monoclonal, 1:1,000 dilution, sc-126) were from Santa Cruz. Rac1 (mouse monoclonal, 1:1,000 dilution, 05-389) and GAPDH (mouse monoclonal, 1:5,000 dilution, MAB374) were from Millipore. P190A was from BD Transduction Laboratories (mouse monoclonal, 1:1,000 dilution, 610149). PIG3 was either from Abcam (mouse monoclonal, 1:1,000 dilution, ab123917) or from Novus Biologicals (mouse monoclonal, 1:100 dilution, NBP2-01301). γ-H2AX (Ser139) was from Abcam (rabbit polyclonal, 1:100 dilution, ab2893). Eight-oxodG was from Trevigen (mouse monoclonal, 1:250 dilution, 4354-MC-050).

Sodium pyruvate (10 mM; SH30239-01) was from Thermo Scientific. Sodium azide (1 mM) was from BDH Lab Supplies (30111). D-Mannitol (20 mM; M4125), N-acetyl-L-cysteine (1 mM; A7250), 4, 5-dihydroxy-1, 3-benzenedisulfonic acid disodium salt monohydrate or Tiron (10 mM; 172553), adriamycin (D1515) and 2-nitrophenyl-β-D-galactopyranoside (N1127) were from Sigma-Aldrich (Saint Louis, USA). H1152 (5μM) and blebbistatin (2.5μM) were from Calbiochem (Nottingham, UK); Y27632 (10μM) and GSK269962 (1μM) were from Tocris Bioscience (Bristol, UK). Fasudil (HA-1077; 10μM) from Selleckchem (Houston, USA).

Plasmids

The RNAi resistant PIG3 construct was obtained by mutagenesis inserting the point mutations A309G, G312A, C315A and C318A with the QuickChange kit (Stratagene, La Jolla, CA, USA) (Fw: 5'-CCCTCTGGGATAGGCATTAGTAGTCCCTCGGGGACAGTGACGTA-3' and Rv: 5'-TACGTCACTGTCCCCGAGGGACTACTAATGCCTATCCCAGAGGG-3') and HA-PIG3 WT as template. Construct was verified by double strand automated sequencing.

PIG3 WT and PIG3 S151V plasmids were kindly provided by Dr Xavier Pares (Universitat Autònoma de Barcelona).

Flag-ARHGAP5 and myc-RhoA plasmids were kindly provided by Prof. Anne Ridley (King´s College London).

Transfection and RNAi

Melanoma cells ($2x10^5$) cells were seeded in a 6-well plate and transfected the next day with 20-40 nM SmartPool or individual OTs (On Target) siRNA oligonucleotides, using Optimem-I and Lipofectamine 2000 (Invitrogen). Forty eight h after transfection, cells were seeded on collagen in 10% FBS, the next day media was changed to 1% FCS (if specified) and cells analysed 24h later. Stable cell lines were selected with 100μg/ml G418 (Sigma). GIPZ Lentiviral shRNA constructs were from Thermo Scientific and stable cells were selected with 1μg/ml puromycin (Life Technologies).

Time-lapse microscopy

Multi-site bright-field microscopy imaging was performed in a humidified chamber at 37°C and 5% CO_2 using a 10×/0.3 NA Plan Fluor ELWD objective lens on a fully motorized (Prior Scientific) multi-field Nikon TE2000 microscope with an ORCA camera (Hamamatsu) controlled by MetaMorph (Molecular Devices) and Volocity (Perkin Elmer) software.

Tracking Assays

Tracking of cell migration within collagen matrices was performed as described (1) over a period of 16h starting after the gel was set. Individual cells were tracked in a semiautomated manner by repeated random selection of cells in movie frames and manual tracing of migration pathways using the Manual Tracking plugin from ImageJ. Speeds are presented as ‘‘box and whisker’’ plots showing median, quartile, and highest and lowest values. Any cells moving through convection were excluded.

Manual classification of cell morphology

Assessment of cell morphology followed the following criteria: for evaluation of the percentage of elongated cells, a cell was considered elongated when its longest dimension was twice the shortest and when it showed at least one long protrusion, whereas cells are classified as rounded when the long and short diameters are approximately equal and protrusions are in the form of blebs.

Quantitative Real Time one step PCR

Trizol (200µl/T12 well) was added to cells seeded on top of thick collagen and collected. RNA was then precipitated and purified using iso-propanol and ethanol precipitation. RNA pellet was resuspended in RNase free water and further purified using spin-easy column purification kit (Sigma). QuantiTect Primer Assays (Qiagen) and Brilliant II SYBR Green QRT-PCR 1-step system (Agilent Technologies) and Stratagene MX 3005p qPCR system were used following the manufacturer’s instructions. GAPDH was used as loading control.

Immunoblotting

Cells on top of thick collagen were lysed in Laemmli Lysis Buffer (containing 1μM DTT, 1mM NaF, 100μM β-glycerol phosphate, 5mM PMSF and Protease inhibitor cocktail tablet from Roche). Lysates were collected, frozen and then boiled for 5min. Samples were sonicated for 15 s prior to centrifugation and supernatants were collected and prepared with LDS loading buffer (Invitrogen). Cell lysates were resolved by SDS-polyacrylamide (PAGE) using pre-made Nu-PAGE™ 4-12% Bis-Tris gels (1.5mm, Invitrogen) and transferred to PVDF filters (0.45μm, Immobilion™). The ECL Plus or Prime ECL detection System (GE Healthcare) with HRP-conjugated secondary antibodies (GE Healthcare) was used for detection. Bands were quantified using Image J (http://rsb.info.nih.gov/ij/).

Rac1-GTP and Rho-GTP pulldown assays

For pulldowns, $2x10^5$cells/well were plated in a 6-well plate and specified treatment was added for the time indicated. Cells were lysed in lysis buffer containing 50mM Tris pH 7.4,10% glycerol, 1% NP40, 5mM $MgCl_2$, 100mM NaCl, 1mM DTT and EDTA free protease inhibitor. Protein lysates were centrifuged at 13000rpm and a small proportion was mixed with LDS loading buffer and stored for determination of total Rac or Rho levels. The remaining protein lysate was incubated with Glutathione S-transferase (GST)-conjugated PAK-CRIB RBD Sepharose beads for 45min or with GST-conjugated Rhotekin RBD Sepharose beads for 30 min. Beads were collected by centrifugation, washed with wash buffer (10mM $MgCl_2$, 1mM DTT, EDTA free protease inhibitor and TBS1x buffer) and resuspended in 20μl 4X LDS loading buffer. Samples were boiled for 5min and supernatants were collected and resolved by SDS-polyacrylamide gels electrophoresis (PAGE).

Quantitative assay of SA-β-gal using cell extracts

SA-β-gal was measured by the rate of conversion of 2-nitrophenyl-β-D-galactopyranoside (ONPG) to O-nitrophenol at pH 6. Equal numbers of cells were collected, washed and resuspended in either 0.4 M sodium acetate-acetic acid buffer (pH 4) or phosphate buffer (pH 6.0). Cells were lysed (10 mM Tris-HCl, 0.5 mM EDTA and 1% Igepal) and the supernatants were mixed with ONPG (2.2 mM),1 mM MgCl2, 50 mM β-mercaptoethanol in either the sodium acetate-acetic acid or phosphate buffer. After incubation at 37 °C for 6 h, two volumes of 0.5 M sodium carbonate were added and absorbance at 420 nm was measured. Values were normalized to protein concentration.

Confocal fluorescent microscopy imaging

Gels were inverted on to MatTek dishes and images were taken with a Zeiss LSM 510 Meta confocal microscope (Carl Zeiss, Germany) with C-Apochromat 40×/1.2 NA (water) objective lense and Zen software (Carl Zeiss). For cell morphology, the shape descriptor “roundness” in ImageJ was used after manually drawing around the cell shape using F-actin staining images. Phospho-MLC2 fluorescence signal was quantified calculating the pixel intensity in single cells relative to the cell area.

For γ-H2AX staining, cells were fixed with 4% p-formaldehyde, permeabilized with 0.5% Triton-X 100 (v/v) and blocked with PBS containing 10% serum, 0.3 M glycine, 1% BSA and 0.1% tween for 2h at room temperature. After blocking, cells were incubated with primary antibody (γ-H2AX, Abcam, ab2893) and with DAPI to stain DNA. Images were taken with a Zeiss LSM 510 Meta confocal microscope (Carl Zeiss, Germany) with C-Apochromat 40×/1.2 NA (water) objective lense and Zen software (Carl Zeiss). Nuclear γ-H2AX fluorescence signal was quantified calculating the pixel intensity in single cell nuclei relative to the nucleus area.

For 8-oxodG staining, cells were fixed with methanol followed by acetone, treated with 0.05N HCl (5min on ice) and with 100µg/ml RNAse for 1h. DNA was denatured *in situ* with 0.15N NaOH in 70% EtOH. After DNA denaturation, treatment with proteinase K was performed.

Coverslips were blocked with 1% BSA for 1h at room temperature and then incubated with primary antibody (8-oxodG, Trevigen, 4354-MC-050), followed by followed by an Alexa 4888-conjugated secondary antibody and with DAPI to stain DNA. Samples were mounted with a medium from DakoCytomation (Carpinteria, CA, USA) and examined with a Leica scanning confocal microscope with 63x objective lense and Leica software. Nuclear 8-oxodG fluorescence signal was quantified calculating the pixel intensity in single cell nuclei relative to the nucleus area.

Intracellular measurement of ROS

Production of intracellular ROS was assessed using 2′, 7′-dichlorodihydrofluorescein diacetate (DCFH-DA, Sigma-Aldrich) a fluorogenic marker for ROS in living cells (2). Briefly, cells were incubated with 20 μM DCFH-DA at 37 °C for 30 min in the dark. After incubation, cells were washed with PBS and fluorescence was analysed with a plate reader (Figure 1B, Figure 1D, Figure 4A and Supplementary Figure 1A), using the excitation peak of fluorescein at 495 nm and the fluorescence emission at 529 nm within 30 min, or by flow cytometry (Figure 4H) using BD FACSCanto™ II systems (BRC Flow Cytometry Core, Guy´s Hospital, London) and FlowJo software.

Annexin V apoptotic assay

To detect apoptotic cells, we used the Annexin V-FITC kit (Miltenyi Biotec) following the manufacture’s instructions. Briefly, cells were incubated with Annexin V-FITC for 15 min in the dark at room temperature, then washed and incubated with propidium iodide (1 μg/ml) prior to analysis by flow cytometry using BD FACSCanto™ II systems (BRC Flow Cytometry Core, Guy´s Hospital, London) and FlowJo software.

3D migration assays

Cells were suspended in serum-free bovine collagen I or rat tail collagen I at 2.3 mg/ml to a final concentration of $10x10^3$ cells/100 μl in a 96-well plate and processed as previously described (3). Cells were mixed with collagen, seeded on a 96-well plate and spun down. 5% FBS-containing media was added on top of the gel after 4h and cells were allowed to invade upwards. After 24h incubation at 37ºC, cells were fixed in 4% formaldehyde for 16 h and stained with 5 μg/ml Hoechst 33258 (Molecular probes-Life Technologies). Plates were imaged on a Zeiss LSM 510 Meta confocal microscope (Carl Zeiss, Germany) with a Plan Apochromat 10×/0.30 objective lenses and Zen software (Carl Zeiss). Images at the bottom at 1μm and at 50μm distance from the bottom were taken. Quadruplicates per condition were used and represented as the average. The 3D migration index was calculated as number of migrating cells at 50 μm/total number of cells.

Immunofluorescence in xenografts

Six μm sections of formalin-fixed, paraffin-embedded material were used. Slides were dewaxed and antigen retrieval was performed using citrate buffer pH 6 followed by blocking in PBS-Tween 0.1% + 1% BSA for 15 min and overnight incubation with mouse monoclonal PIG3 antibody (1:100 in PBS+1% BSA). Antibody detection was performed using the Liquid Permanent Red Chromogen (Dako). Hematoxylin was used to counterstain. Quantification of cell morphology (roundness using ImageJ) was performed on H&E stainings. Each mouse xenograft was imaged for 6 separate fields and 10 representative cells were scored for roundness index. The average cell rounded index per field was plotted in Figure 7C (right panel).

RNAi sequences

<u>Sh plasmid sequences</u>

shPIG3-86

TCTAGAATAAGATTAACTC

shPIG3-14

TTCCCACAAGATGTAACAG

siRNA sequences

ARHGAP5-#1, SmartPool

GAGAGCAGAUUUCCAGUUA

UGAGAUCACUGCUAAAUUU

GGCUAAAGUGAGUUGAUAA

CUUCAUAGUGAGUCAUUUA

ARHGAP5-#2

GCUGAUACAACCACAAUUAUU

p190A-SmartPool

GGAGGAAUCUGUAUACAUG

GAACAGCGAUUUAAAGCAU

GAUGGGCUGUCUUUCAUUA

UCAGCGAGAUCCAAUGUAA

PIG3-#1

GAAGGGCUCCUCAUGCCUA

PIG3-#2

GGAUUUCUCUGAAGCAACG

PIG3-#3, SmartPool

GGGAGGAGGUGACAUCAAU

GGUCUAGGGACAAUAAGUA

GCAGAGACAAGGCCAGUAU

GAGACUAUGUGCUAAUCCA

Rac1, SmartPool

CGGCACCACUGUCCCAACA

AUGAAAGUGUCACGGGUAA

UAAAGACACGAUCGAGAAA

UAAGGAGAUUGGUGCUGUA

Supplementary References

1. Wilkinson S, Paterson HF and Marshall CJ. Cdc42-MRCK and Rho-ROCK signalling cooperate in myosin phosphorylation and cell invasion. Nat Cell Biol. 2005; 7, 255-261.
2. Royall JA, Ischiropoulos H. Evaluation of 2',7'-dichlorofluorescin and dihydrorhodamine 123 as fluorescent probes for intracellular H2O2 in cultured endothelial cells. *Archives of biochemistry and biophysics.* 1993;302(2):348-355.
3. Sanz-Moreno V, Gadea G, Ahn J, et al. Rac activation and inactivation control plasticity of tumor cell movement. *Cell.* 2008;135(3):510-523.

Supplementary Tables

Supplementary Table 1, related to Figure 2.

Gene set enrichment analysis (GSEA) analysis: gene sets significantly enriched in ROCK-inhibited A375M2 melanoma cells.

Biological category	Gene set	Size	NES*	Nominal p-value
**ROS metabolism**	HOUSTIS_ROS	30	-1.49	0.046
	GO_ROS	26	-1.54	0.04
	ELECTRON_TRANSPORTER_ACTIVITY	113	-1.4	0.018
	ELECTRON_TRANSPORT_GO_0006118	50	-1.49	0.017
	OXIDOREDUCTASE_ACTIVITY__ACTING_ON_CH_OH_GROUP_OF_DONORS	60	-1.45	0.036
	OXIDOREDUCTASE_ACTIVITY	277	-1.26	0.037
	OXIDOREDUCTASE_ACTIVITY_GO_0016616	54	-1.44	0.026
	HSA00980_METABOLISM_OF_XENOBIOTICS_BY_CYTOCHROME_P450	60	-1.57	0.005
	HSA00590_ARACHIDONIC_ACID_METABOLISM	52	-1.54	0.008
**DNA damage**	UVC_TTD_4HR_UP	58.000	-1.820	0.000
	DOX_RESIST_GASTRIC_UP	44	-1.82	0.004
	GENOTOXINS_ALL_4HRS_REG	26	-1.47	0.041
**Antioxidant activity**	GLUTATHIONE_METABOLISM	31	-1.73	0.002
	HSA00480_GLUTATHIONE_METABOLISM	37	-1.51	0.039

(*) NES stands for Normalized Enrichment Score.

Supplementary Table 2, related to Figure 2.

Gene set enrichment analysis (GSEA) analysis: gene sets significantly enriched in A375P cells compared to A375M2 cells.

	Biological category	Gene set	Size	NES	Nominal p-value
	**ROS metabolism**	HOUSTIS_ROS	30	-1.64	0.018
		GO_ROS	26	-1.62	0.026
		ELECTRON_TRANSPORTER_ACTIVITY	113	-1.46	0.007
		OXIDOREDUCTASE_ACTIVITY	277	-1.20	0.039
	**DNA damage**	UVC_TTD_4HR_UP	58.000	-1.43	0.03
	**Antioxidant activity**	GLUTATHIONE_METABOLISM	31	-1.6	0.015
		ANTIOXIDANT_ACTIVITY	18	-1.58	0.034

(*) NES stands for Normalized Enrichment Score.

Supplementary Figures

Supplementary Figure 1, related to Figure 1.

A) ROS measurement of A375M2 cells depleted for Rac1 by siRNA transfection prior to incubation with the ROCK inhibitor Y27632 (10μM) for 24h. ROS levels are represented as relative mean fluorescence (n = 4, error bars are mean ±SD, two-sided Student's t test was used to generate p values, **p< 0.01). A representative immunoblot of Rac1 levels is shown on the right. **B**) Schematic diagram representing the enzymatic reactions from the molecule of oxygen leading to the generation of the different reactive oxygen species. MPO stands for myeloperoxidase, SOD for superoxide dismutases, XO for xanthine oxidases and COX for cyclooxigenases. **C**) Representative immunoblot (**top**) and quantification (**bottom**) of Rac1-GTP in pulldown samples and of total Rac1 in total lysate of H_2O_2 treated A375M2 cells (n = 7, error bars are ±SD, two-sided Student´s t test was used to generate p values, *p<0.05, **p<0.01). **D**) Cell morphology of A375P cells treated with the ROS scavengers N-acetyl cysteine (N-Ac-cysteine), mannitol, sodium azide, tiron and sodium pyruvate for 24h on top of atelopeptide bovine collagen I (n = 4, error bars are ±SD, two-sided Student´s t test was used to generate p values, *p<0.05, **p<0.01, ****p<0.0001). **E**) Cell morphology of A375P cells treated with the ROS scavengers N-acetyl cysteine, mannitol, sodium azide, tiron and sodium pyruvate on top of atelopeptide bovine collagen I according to 'roundness' factor (ImageJ classification): closer to zero more elongated; closer to 1 more rounded. Dots represent single cells from three independent experiments. IF stands for immunofluoresence (n = 3 experiments; N = 90 cells, error bars are ±SD, two-sided one-way ANOVA with Tukey´s *post hoc* test was used to generate p values, ****p<0.0001). **F**) Quantification of p-MLC2 fluorescence signal relative to the cell area of confocal images in A375P cells after incubation with the ROS inhibitors N-acetyl cysteine, mannitol, sodium azide, tiron and sodium pyruvate on collagen I. Dots represent single cells from three independent experiments and values are normalized to the cell area (n = 3 experiments; N = 90 cells, error bars are ±SD, two-sided one-way ANOVA with Tukey´s *post hoc* test was used to generate p values, *p<0.05, ***p<0.001, ****p<0.0001). **G**) Representative confocal images

of p-MLC2 (green) and F-actin immunostaining (red) in A375P cells treated with the ROS scavengers N-acetyl cysteine, mannitol, sodium azide, tiron and sodium pyruvate for 24h on atelopeptide bovine collagen I. Inset shows cell blebbing in all treatments. Scale bar, 20 μm.

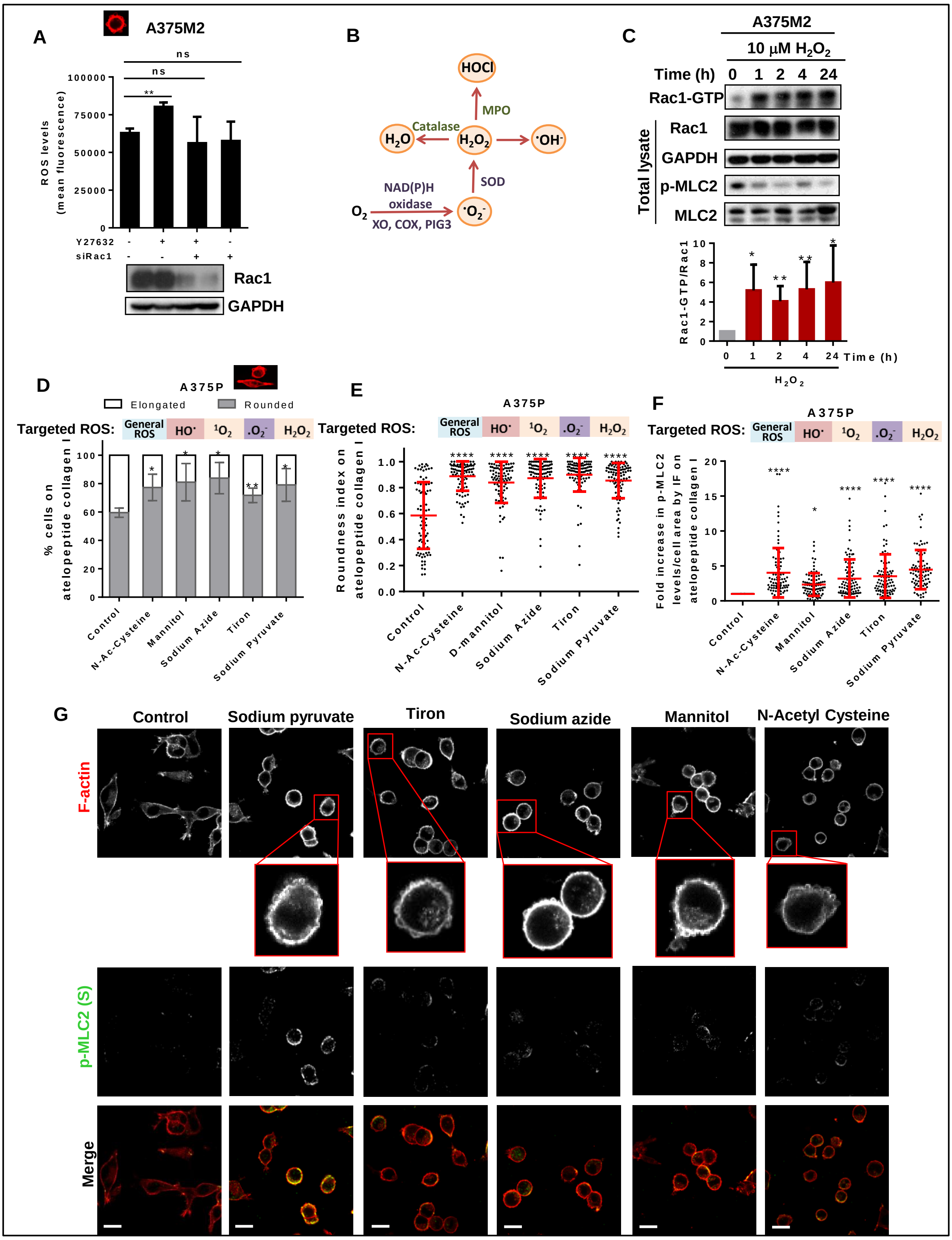

Supplementary Figure 2, related to Figure 2.

A) Gene set enrichment analysis (GSEA) of a previous gene expression microarray analysis between ROCK-inhibited A375M2 cells and untreated A375M2 cells. Enrichment plots showed up-regulation of genes related to DNA damage and ROS processes in elongated-less contractile ROCK-inhibited A375M2 cells compared to A375M2 cells (nominal p values are shown on each gene set). **B**) Quantification of p-MLC2, p-ATM, p-p53 (S15) and p53 levels in A375M2 after inhibiting ROCK with H1152 for 4, 24 and 48h in A375M2 (n = 5, error bars are ±SD, two-sided Student´s t test was used to generate p values, *p<0.05, *p<0.01, ** p<0.001). **C**) Quantification of p-MLC2, p-ATM, p-p53 (S15) and p53 levels in A375M2 compared to A375P cells (n = 5, error bars are ±SD, two-sided Student´s t test was used to generate p values, *p<0.05, **p<0.01, ***p<0.001). **D**) Quantification of p-MLC2 (top) and p53 levels (bottom) in A375M2 cells treated with ROCK inhibitors H1152, Y27632 and Fasudil, ROCK inhibitor GSK269962 and contractility inhibitor blebbistatin (n = 5, error bars are ±SD, two-sided one-way ANOVA with Tukey´s *post hoc* test was used to generate p values, *p<0.05, **p<0.01, ***p<0.001, ****p<0.0001). **E**) Quantification of the nuclear levels in arbitrary units (a.u.) of γ-H2AX in A375M2 after treatment with Adriamycin for 12h (0.25 μg/ml). Ctr stands for control (n = 3 experiments; N=150 cells, error bars are ±SD, two-sided one-way ANOVA with Tukey´s *post hoc* test was used to generate p values, ****p<0.0001). **F**) Quantification of the nuclear levels in arbitrary units (a.u.) of 8-oxodG in A375M2 after treatment with Adriamycin for 12h (0.25 μg/ml). Ctr stands for control (n = 3 experiments; N = 150 cells, error bars are ±SD, two-sided one-way ANOVA with Tukey´s *post hoc* test was used to generate p values, ****p<0.0001). **G**) Quantification (**top**) and representative immunoblot (**bottom**) of PIG3 levels in A375M2 cells after ROCK inhibition with H1152 (5μM) for the times indicated (n = 3, error bars are ±SD, two-sided Student´s t test was used to generate p values, *p<0.05, **p< 0.01). **H**) PIG3 protein levels in A375M2 cells after ROCK inhibitor Y27632 (10μM) treatment for 24h. Cell morphology and a representative

immunoblot for PIG3 are shown below (n = 3, error bars ±SD, two-sided Student´s t test was used to generate p values, *p<0.05).

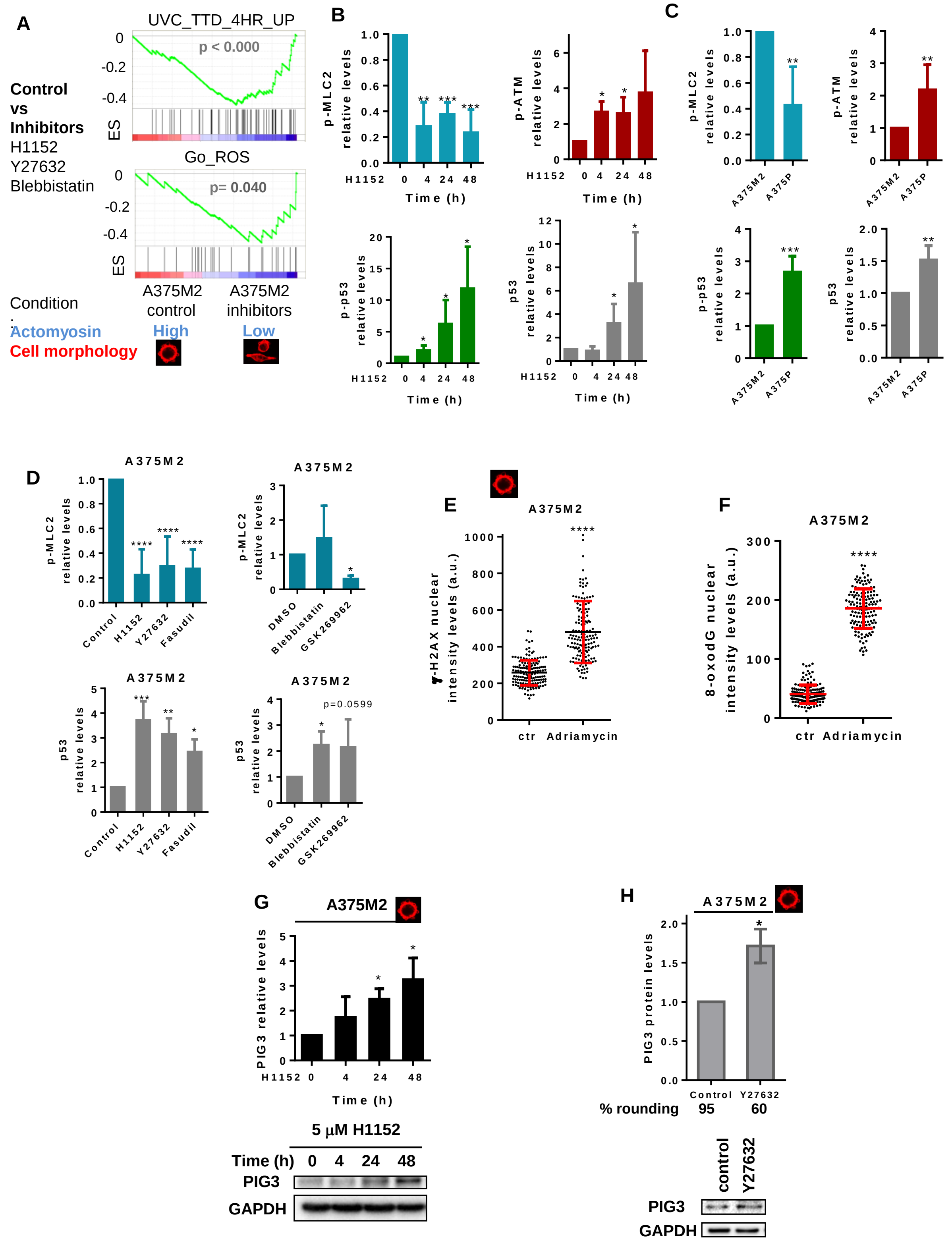

Figure S2

Supplementary Figure 3, related to Figure 3.

A) Representative bright-field images of WM1361, SBCL2 and A375P cells on top of bovine collagen I after PIG3 depletion using a smart pool (#3) of oligonucleotides. Scale bar, 50 μm. **B**) Cell morphology (roundness) of A375P cells on top of bovine collagen I after PIG3 knockdown (siPIG3). Dots represent single cells from more than three independent experiments (n = 3 experiments; N = 60 cells, error bars are ±SD, two-sided one-way ANOVA with Tukey´s *post hoc* test was used to generate p values, ****p<0.0001). **C**) Representative images showing the increase in cell rounding in A375P cells upon p53 knockdown. Scale bar, 50 μm. **D**) Quantification of p-MLC2 levels (left) and p53 and PIG3 protein levels (middle) in p53-ablated A375P cells. Representative immunoblots for p53 and PIG3 are shown on the right panel (n = 3, error bars ±SD, two-sided Student´s t test was used to generate p values, *p<0.05). **E**) Quantification of p-MLC2 levels in A375P cells overexpressing empty vector or RNAi resistant PIG3 prior to PIG3 knockdown (n = 3, error bars are ±SD, two-sided Student´s t test was used to generate p values, *p<0.05). **F**) Quantification of p-MLC2 levels in A375P cells after depletion of PIG3 by shRNA. Percentage of knockdown (KD) is shown above (n = 3, error bars are ±SD, two-sided one-way ANOVA with Tukey´s *post hoc* test was used to generate p values, *p<0.05, **p<0.01).

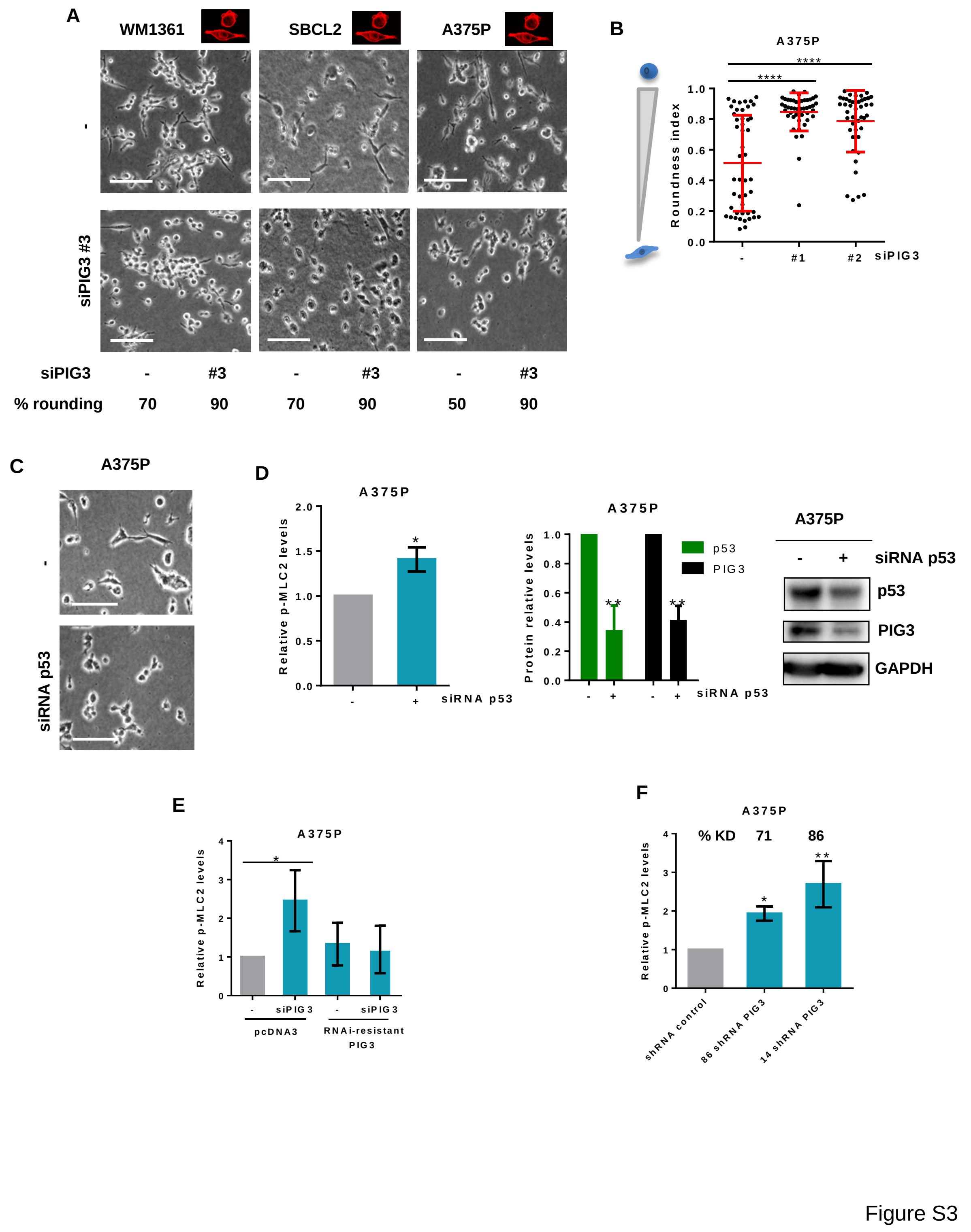
A
WM1361
SBCL2
A375P
-
siPIG3 #3
siPIG3
% rounding
70
90
70
90
50
90
B
A375P
****
****
Roundness index
siPIG3
#1
#2
C
A375P
siRNA p53
D
A375P
Relative p-MLC2 levels
siRNA p53
Protein relative levels
p53
PIG3
GAPDH
E
A375P
Relative p-MLC2 levels
siPIG3
pcDNA3
RNAi-resistant PIG3
F
A375P
% KD
71
86
Relative p-MLC2 levels
shRNA control
86 shRNA PIG3
14 shRNA PIG3

Figure S3

Supplementary Figure 4, related to Figure 4.

Dot plots (**left**) and percentage of apoptotic cells (**right**) obtained from Annexin V-FITC and propidium iodide (PI) apoptotic assay of A375M2 cells overexpressing empty vector pcDNA3, PIG3 WT or PIG3 S151V or treated with adriamycin (0.5 μg/ml) for 12h. Each dot plot consists of data showing live cells (FITC and PI negative, bottom left square), early apoptotic population (FITC positive, bottom right square) and mid-late stage apoptosis (FITC positive and PI positive, top right square). Percentage of apoptotic cells is shown in the dot plots (n = 4, error bars are ±SD, two-sided Student´s t test was used to generate p values, *p< 0.05).

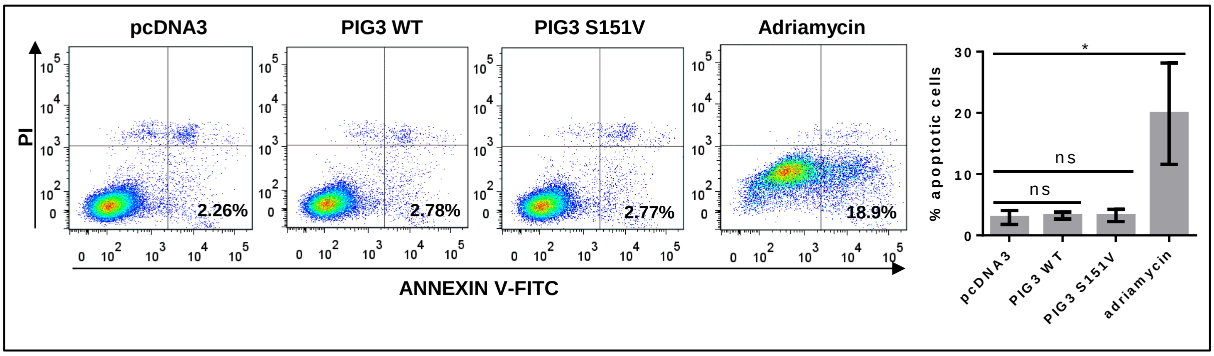

Supplementary Figure 5, related to Figure 5.

A) Representative bright-field images (**left**) and cell morphology (percentage of rounded and elongated cells, **right**) of A375M2 cells transfected with siRNA for p190A-RhoGAP, seeded on atelopeptide bovine collagen I and 72h post-transfection, treated with H_2O_2 (10μM) for 24h. Scale bar, 20 μm (n = 3, error bars are ±SD, two-sided one-way ANOVA with Tukey´s *post hoc* test was used to generate p values, $**p<0.01$, $***p<0.001$). Immunoblot for p190A-RhoGAP is shown on the right. **B**) Representative confocal images (**left**) of p-MLC2 (green) immunofluorescence (IF) stainings and quantification of p-MLC2 (Ser19) levels (**right**) of ARHGAP5-ablated A375M2 cells stimulated with H_2O_2 (10μM) for 24h. F-actin was also stained (red). Each dot represents a single cell, values are relative to the cell area and represented in arbitrary units (a.u.) (n = 3 experiments; N = 90 cells, error bars are ±SD, two-sided one-way ANOVA with Tukey´s *post hoc* test was used to generate p values, $*p<0.05$, $****p<0.0001$). Scale bar, 20 μm. **C**) Cell morphology of A375P and WM1361 cells transfected with individual or a pool of oligonucleotides (#1 and #2) to knockdown ARHGAP5 expression on atelopeptide bovine collagen I. Knockdown (KD) levels are shown below as % (n = 3, error bars are ±SD, two-sided Student's t test was used to generate p values, $*p<0.05$, $**p< 0.01$, $***p<0.001$). **D**) Representative immunoblots of p-MLC2 in ARHGAP5-abrogated A375P and WM1361 cells on atelopeptide bovine collagen I. Knockdown (KD) levels are shown below as % (n = 3, representative immunoblots are shown). **E**) Quantification of p-MLC2 levels of A375P and WM1361 cells after depletion of ARHGAP5. Knockdown (KD) levels are shown below as % (n = 5, error bars are ±SD, two-sided Student's t test was used to generate p values, $*p<0.05$). **F**) Representative confocal images of PIG3 (blue) immunostainings of A375M2 cells on atelopeptide bovine collagen I transfected with siRNA for ARHGAP5 and overexpressing wild-type PIG3. F-actin was also stained (red). Scale bar, 10 μm. **G**) Cell morphology of A375M2 cells transfected with empty vector or PIG3 WT and with a pool of oligonucleotides for ARHGAP5 depletion and plated on

atelopeptide bovine collagen I (n = 3, error bars are ±SD, two-sided one-way ANOVA with Tukey´s *post hoc* test was used to generate p values, ****p<0.0001).

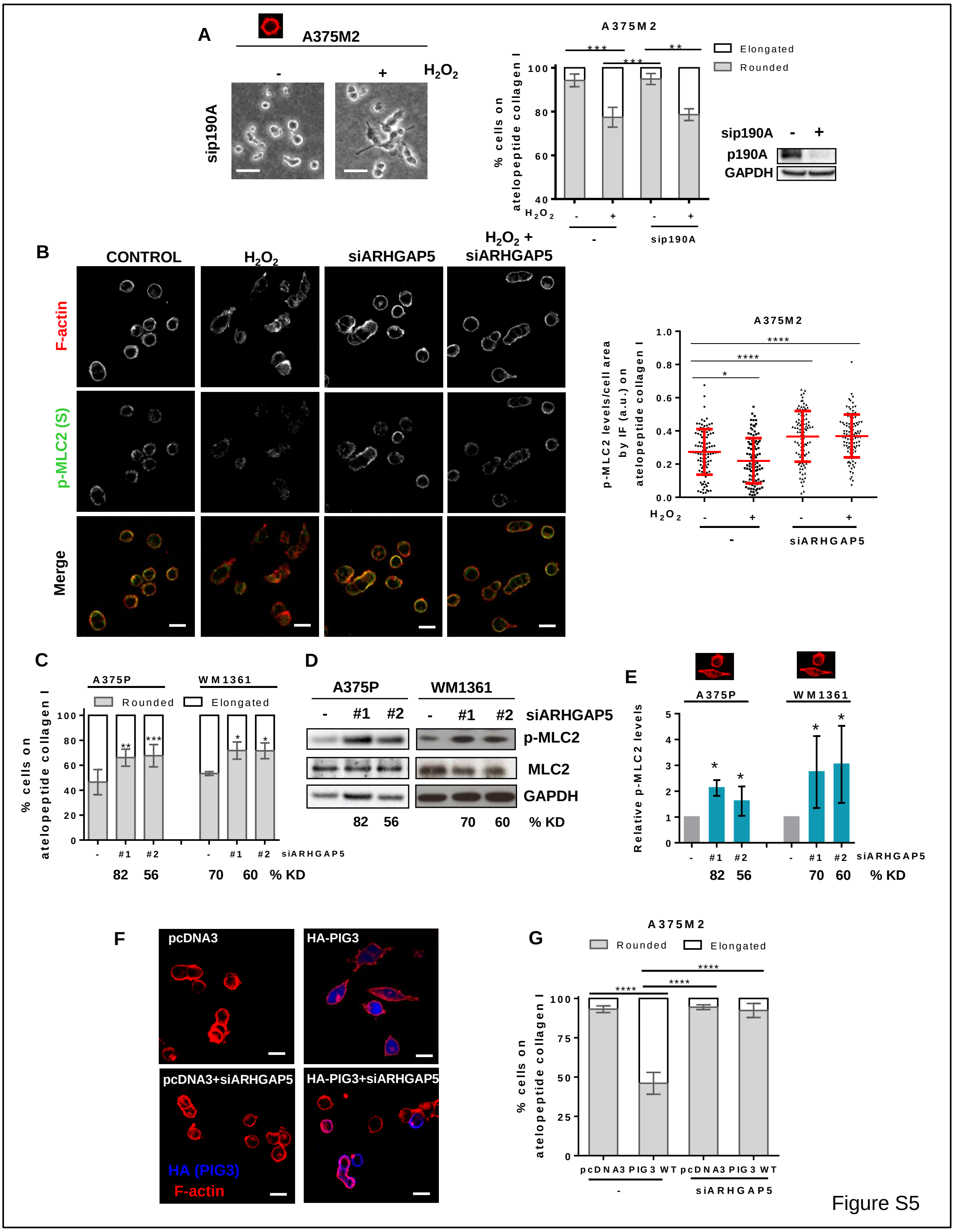

Figure S5

Supplementary Figure 6, related to Figure 7.

A) Representative confocal images of PIG3 and GFP-stably transfected A375M2 cells on a bovine collagen I. Scale bar, 20 μm. **B**) Cell morphology of A375M2 cells overexpressing PIG3 (n = 3, error bars are ±SD, two-sided Student´s t test was used to generate p values). **C**) Immunoblots for p-MLC2 and PIG3 protein levels in A375M2 stably transfected with PIG3 (n = 4, a representative experiment is shown). **D**) Quantification of p-MLC2 levels in PIG3 overexpressing A375M2 cells on collagen I (n = 4, error bars are ±SD, two-sided Student´s t test was used to generate p values, ****p<0.0001). **E**) Quantification of the number of motile cells in control and in PIG3 WT overexpressing A375M2 cells (7 fields/mouse; 7 mice/condition, error bars are ±SD, two-sided Student's t test was used to generate p values). In this graph, streaming was quantified as amoeboid. **F**) Representative images of xenografts of mouse tumors of A375M2 control or PIG3 WT over-expressing cells stained for PIG3. Scale bar, 1mm (7 mouse xenografts/condition).

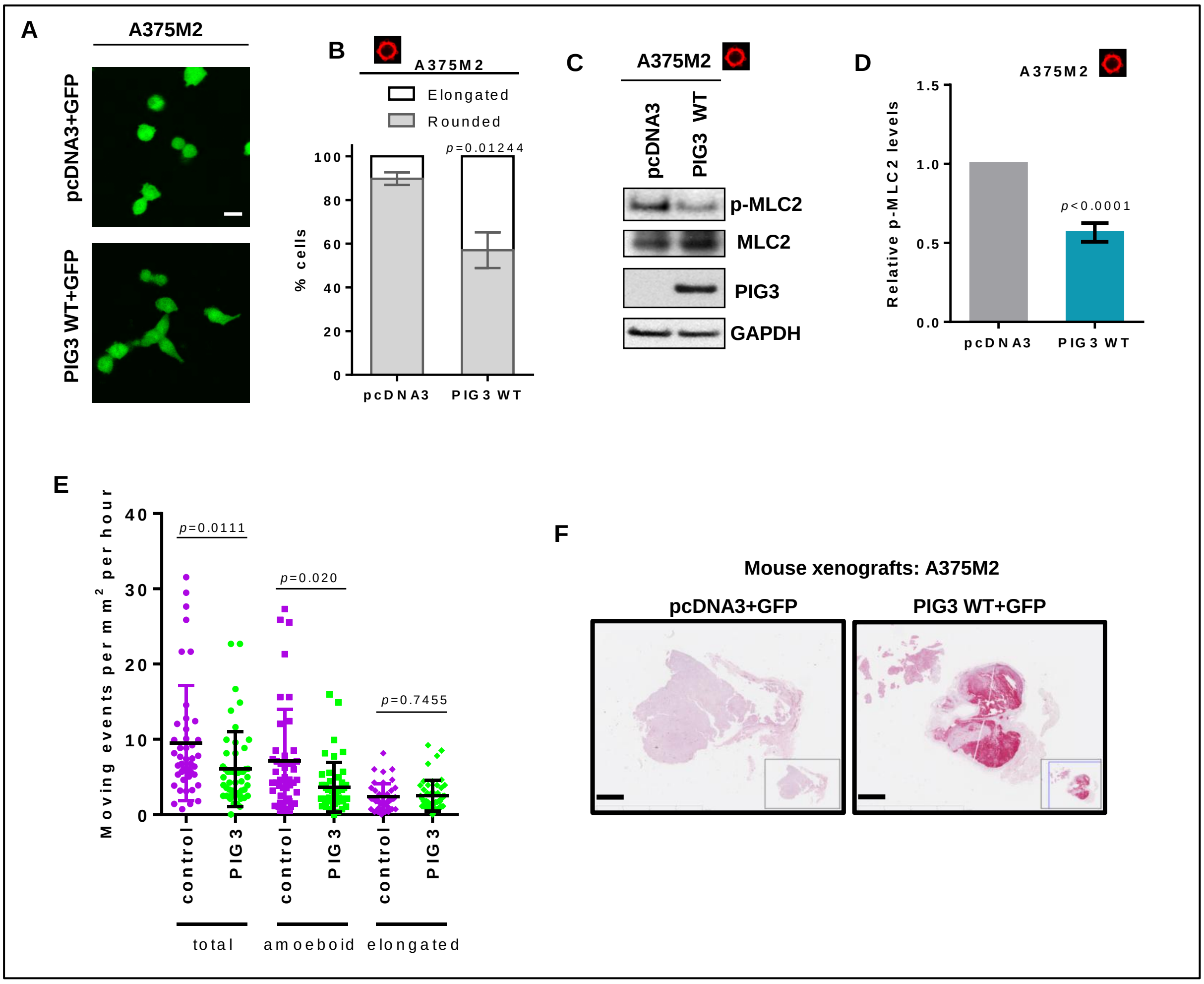

A
A375M2
pcDNA3+GFP
PIG3 WT+GFP
B
A375M2
Elongated
Rounded
p=0.01244
% cells
100
80
60
40
20
0
pcDNA3
PIG3 WT
C
A375M2
pcDNA3
PIG3 WT
p-MLC2
MLC2
PIG3
GAPDH
D
A375M2
Relative p-MLC2 levels
1.5
1.0
0.5
0.0
p<0.0001
pcDNA3
PIG3 WT
E
Moving events per mm2 per hour
40
30
20
10
0
p=0.0111
p=0.020
p=0.7455
control
PIG3
control
PIG3
control
PIG3
total
amoeboid
elongated
F
Mouse xenografts: A375M2
pcDNA3+GFP
PIG3 WT+GFP

Supplementary Figure 7, related to Figure 8.

A) PIG3, ROCK1 and ROCK2 expression in primary and metastasis melanoma human tissues using z-scores for mRNA expression data from TCGA (error bars are ±SD, Mann–Whitney test was used to generate p values, *** p<0.001, ****p<0.0001). B) Scatter plots of PIG3, ROCK1 and ROCK2 expression correlation analysis (Pearson´s r) using normalized mRNA expression data from TCGA database of primary melanomas (**top panels**) and metastatic tumours (**bottom panels**) Results showed a significant negative correlation between PIG3 and ROCK1 expression (r = -0.3823 for primary tumours and r = -0.1590 for metastasis). A negative correlation was also observed between PIG3 and ROCK2 expression (r = -0.1518 for primary tumours and r = -0.06665 for metastasis) although the correlation analysis in primary tumours and metastasis separately did not reach significant results. Analysis showed a significant positive correlation between ROCK1 and ROCK2 expression (r = 0.8016 for primary tumours and r = 0.6357 for metastasis).

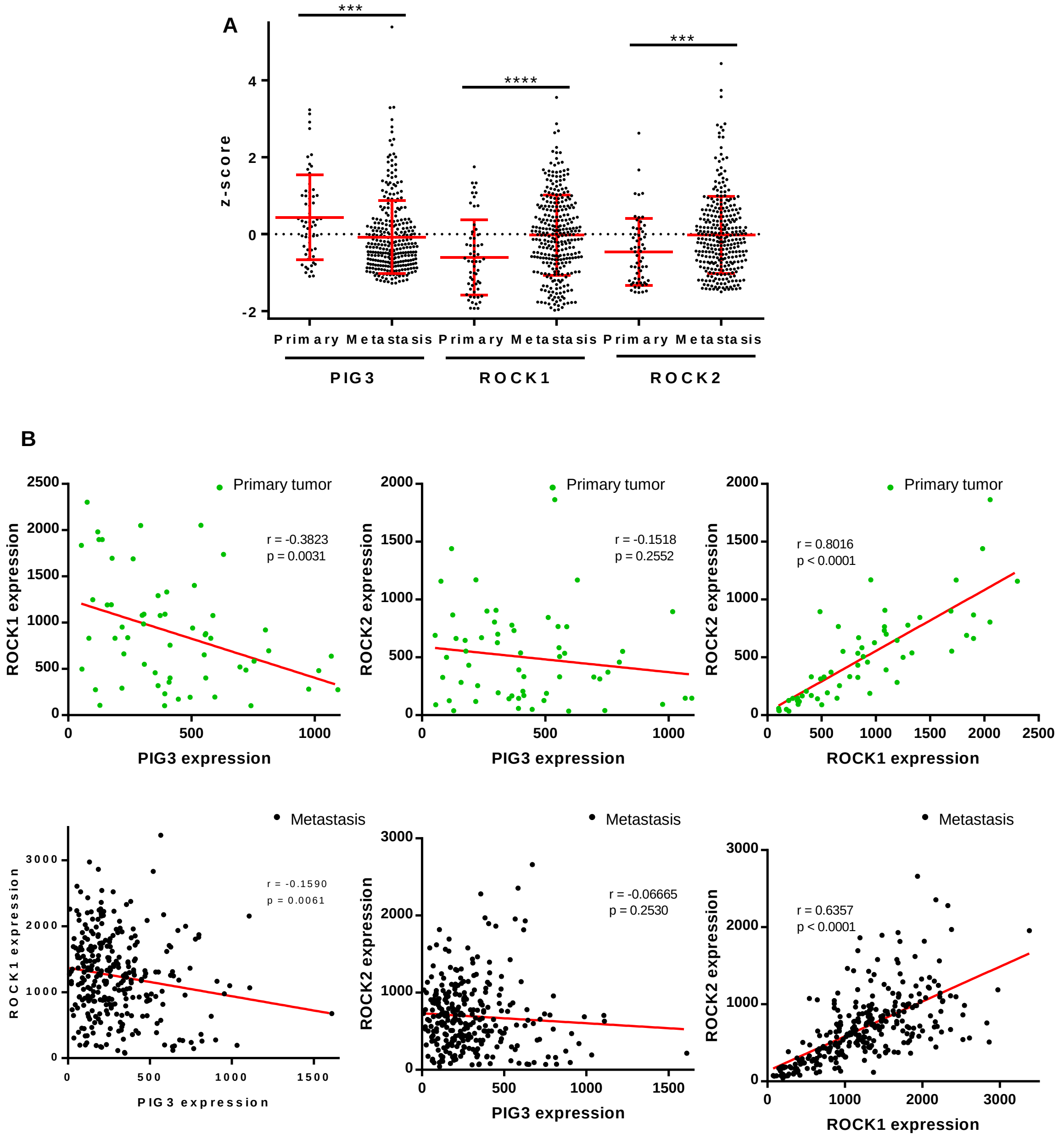

A
z-score
***
****
***
Primary Metastasis Primary Metastasis Primary Metastasis
PIG3
ROCK1
ROCK2
B
Primary tumor
r = -0.3823
p = 0.0031
ROCK1 expression
PIG3 expression
Primary tumor
r = -0.1518
p = 0.2552
ROCK2 expression
PIG3 expression
Primary tumor
r = 0.8016
p < 0.0001
ROCK2 expression
ROCK1 expression
Metastasis
r = -0.1590
p = 0.0061
ROCK1 expression
PIG3 expression
Metastasis
r = -0.06665
p = 0.2530
ROCK2 expression
PIG3 expression
Metastasis
r = 0.6357
p < 0.0001
ROCK2 expression
ROCK1 expression

Figure S7

Supplementary Figure 8, related to Discussion.

A) Representative immunoblots (left) for p53, PIG3 and pMLC2 and quantification (right) for p53 and PIG3 protein levels after treatment with cytotoxic drug, adriamycin (0.25μg/ml) and with the ROCK inhibitor H1152 (5μM) for 24h in A375M2 cells. Quantification levels are represented as fold increase relative to control (n = 8, error bars ±SD, two-sided Student´s t test was used to generate p values, *p<0.05, **p<0.01, ***p<0.001). **B**) Quantification of p53 levels in mutant TP53 melanoma 501MEL (p53 C277W) and Skmel28 (p53 L145R) cells after treatment with ROCK inhibitor H1152 (5μM) for 24h. Ctr stands for control (n = 5, error bars are ±SD). **C**) Enzymatic activity of lysosomal β-galactosidase (pH 4, left panel) and senescence-associated β-galactosidase (pH 6, right panel) in A375M2 overexpressing empty vector or WT PIG3 (n = 4, error bars are ±SD, two-sided Student´s t test was used to generate p values, ***p<0.001).

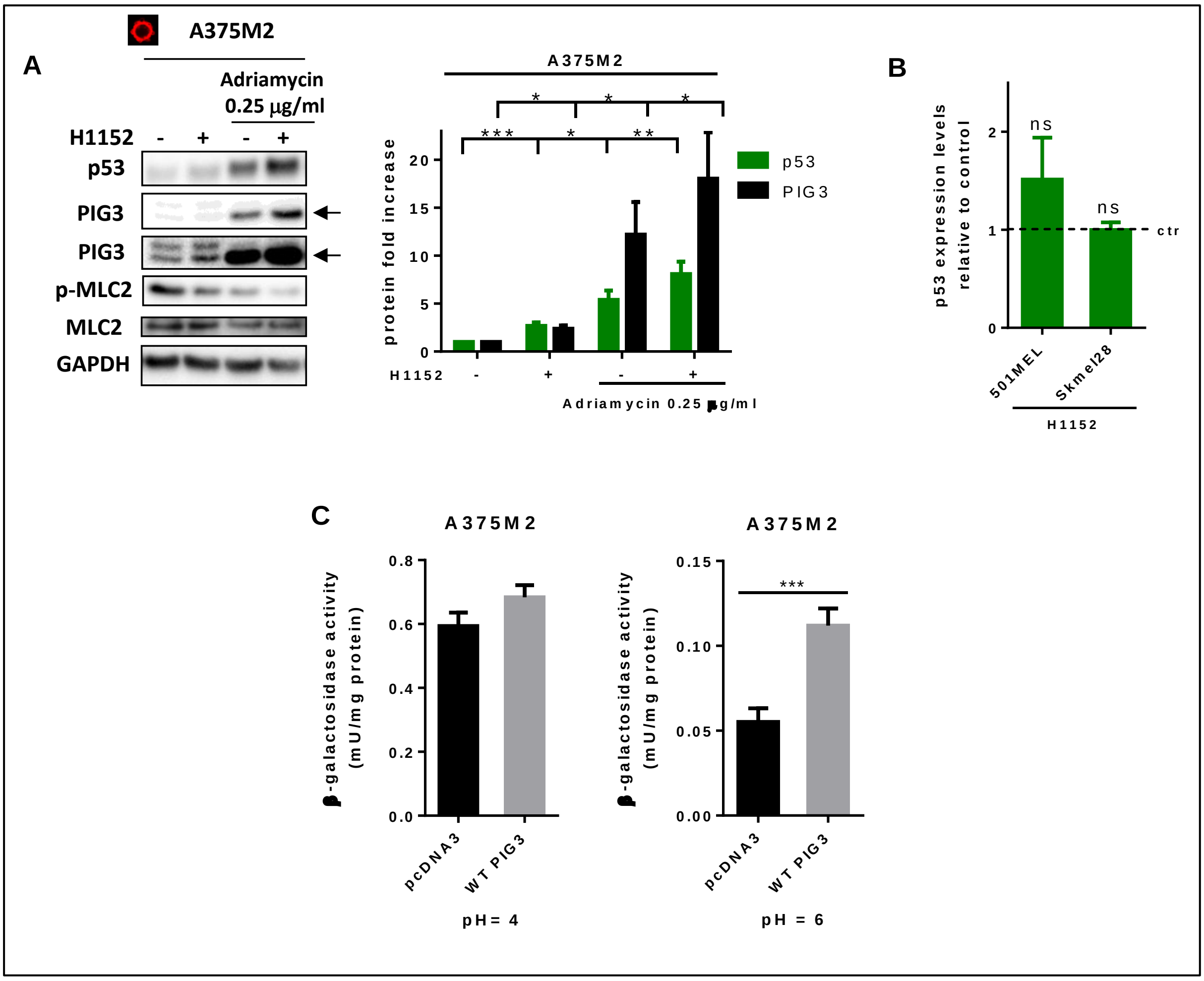

Supplementary Movies

Supplementary Movie 1 and 2, related to Figure 3.

Time-lapse video microscopy with a 10x magnification objective of A375P less contractile cells stably transfected with shRNA control (Movie 1) compared to shRNA against PIG3 (Movie 2) and seeded on atelopeptide bovine collagen I.

Supplementary Movie 3 and 4, related to Figure 7.

Time-lapse video microscopy with a 10x magnification objective of A375M2 very contractile cells stably transfected with GFP and either empty vector pcDNA3 (Movie 3) or PIG3 WT (Movie 4) and seeded on a thick layer of atelopeptide bovine collagen I.

Supplementary Movie 5 and 6, related to Figure 7.

In vivo two-photon microscopy imaging of subcutaneous tumours grown in mice. GFP-A375M2 cells transfected with empty vector (Movie 5) and GFP-A375M2 cells over-expressing PIG3 WT (Movie 6) were imaged. Control cells display fast amoeboid movement into the matrix in the cortical regions, whereas in PIG3 overexpressing tumors there is a reduction of tumor cell motility.
